# Eugenol specialty chemical production in transgenic poplar (*Populus tremula *×* P. alba*) field trials

**DOI:** 10.1111/pbi.12692

**Published:** 2017-03-07

**Authors:** Da Lu, Xianghe Yuan, Sung‐Jin Kim, Joaquim V. Marques, P. Pawan Chakravarthy, Syed G. A. Moinuddin, Randi Luchterhand, Barri Herman, Laurence B. Davin, Norman G. Lewis

**Affiliations:** ^1^ Institute of Biological Chemistry Washington State University Pullman WA USA; ^2^ Puyallup Research and Extension Center Washington State University Puyallup WA USA

**Keywords:** Eugenol, hybrid poplar, phytochemical factories, phenylpropanoid metabolism

## Abstract

A foundational study assessed effects of biochemical pathway introduction into poplar to produce eugenol, chavicol, *p*‐anol, isoeugenol and their sequestered storage products, from potentially available substrates, coniferyl and *p*‐coumaryl alcohols. At the onset, it was unknown whether significant carbon flux to monolignols vs. other phenylpropanoid (acetate) pathway metabolites would be kinetically favoured. Various transgenic poplar lines generated eugenol and chavicol glucosides in *ca*. 0.45% (~0.35 and ~0.1%, respectively) of dry weight foliage tissue in field trials, as well as their corresponding aglycones in trace amounts. There were only traces of any of these metabolites in branch tissues, even after ~4‐year field trials. Levels of bioproduct accumulation in foliage plateaued, even at the lowest introduced gene expression levels, suggesting limited monolignol substrate availability. Nevertheless, this level still allows foliage collection for platform chemical production, with the remaining (stem) biomass available for wood, pulp/paper and bioenergy product purposes. Several transformed lines displayed unexpected precocious flowering after 4‐year field trial growth. This necessitated terminating (felling) these particular plants, as USDA APHIS prohibits the possibility of their interacting (cross‐pollination, etc.) with wild‐type (native plant) lines. In future, additional biotechnological approaches can be employed (e.g. gene editing) to produce sterile plant lines, to avoid such complications. While increased gene expression did not increase target bioproduct accumulation, the exciting possibility now exists of significantly increasing their amounts (e.g. 10‐ to 40‐fold plus) in foliage and stems via systematic deployment of numerous ‘omics’, systems biology, synthetic biology and metabolic flux modelling approaches.

## Introduction

**Figure 1:**
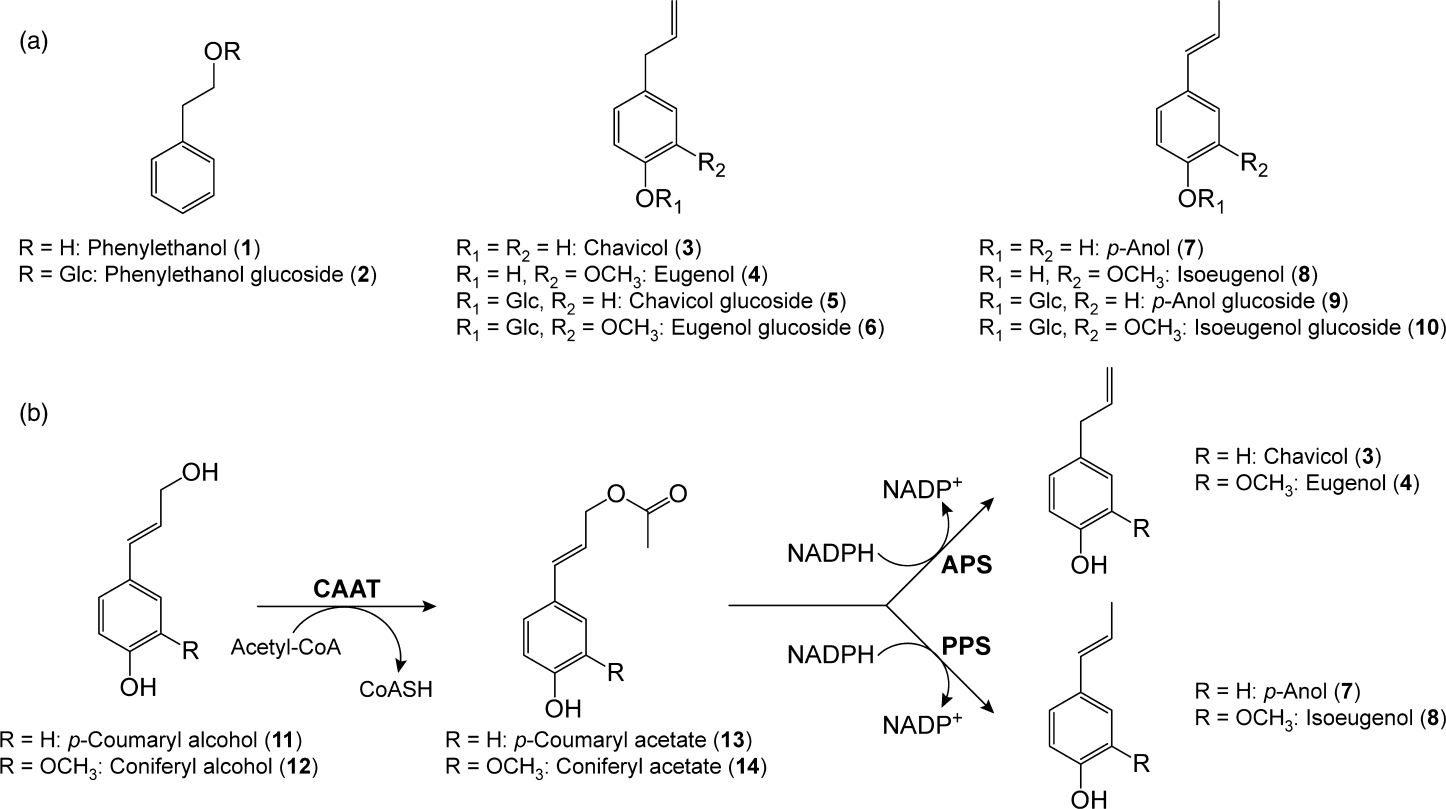
Phenyl derivatives and monolignol conversions to allyl/propenylphenols. (a) Phenylethanol (**1**), phenylethanol glucoside (**2**), allyl/propenylphenols **3**,** 4**,** 7** and **8** and allyl/propenylphenol glucosides **5**,** 6**,** 9** and **10**. (b) Biosynthetic pathway to allyl/propenylphenols **3**,** 4**,** 7** and **8**. APS, allylphenol synthase; CAAT, cinnamyl alcohol acyltransferase; PPS, propenylphenol synthase.

Naturally occurring tree species worldwide have long been used by humanity as major sources of wood products, pulp, paper, other polymers (e.g. natural rubber), resins and oils, spices, fragrances, perfumes, various specialty chemicals, medicinals and numerous foodstuffs, such as fruits and nuts (Patten *et al*., 2010). More recently, through genetic engineering, hybrid poplar (*Populus tremula *×* P. alba*) was also demonstrated able to produce the characteristic rose oil constituent, phenylethanol (**1**, Figure 1a), as a specialty chemical in amounts potentially suitable for commercial production. The phenylethanol (**1**) accumulating was largely sequestered *in vivo* as its phenylethanol glucoside (**2**), rather than in its aglycone form (Costa *et al*., 2013a,b; Doughton, 2014).

The U.S. Congress has stipulated that, by 2030, 30% of petroleum‐derived gasoline is to be replaced by renewables from lignocellulosic plant/algae sources (Houghton *et al*., 2006). Yet, currently, the key technical barriers to massively utilize lignocellulosic feedstocks for liquid fuels have not been overcome so as to be economically competitive. Much attention is thus now being increasingly given to developing the means to economically produce value‐added bioenergy crops with valuable bioproducts, in order to meet—if not exceed—congressional targets. That is, there is an increasing emphasis on producing bioproducts, as well as lignocellulosic biomass, to enable future biorefineries to be economically competitive.

Among various plant species considered for such purposes, hybrid poplar has many advantages including: rapid growth and development; ease of biological transformation (e.g. for introducing new biochemical pathways as above or for reducing lignocellulosic recalcitrance); facile harvesting of plant material through coppicing (e.g. perhaps three harvests per 2 years growth in ultrashort rotations), as well as for its considerable potential as a bioenergy crop suitable for deployment in marginal lands (i.e. land not used for food production). An additional potential advantage for designing future genetic manipulations (including gene editing) with hybrid poplar is that its close relative *P. trichocarpa* has had its genome sequenced (Tuskan *et al*., 2006).

In the current study, genetic engineering of poplar was envisaged to mainly produce bioproducts, eugenol (**4**), isoeugenol (**8**) or homologs/derivatives thereof via heterologous introduction of genes encoding their pathways from coniferyl alcohol (**12**) (Figure 1b) (Kim *et al*., 2014). Eugenol (**4**) was of particular interest, as it is a major constituent of the clove tree (*Syzygium aromaticum*, or *Eugenia caryophyllata*), where it largely accumulates in cloves and clove oil [with the latter up to 89% eugenol (**4**)] (Chaieb *et al*., 2007). Moreover, cloves and clove oil have been highly valued in the West since the Age of Exploration, when maritime trade routes connected European consumers to spice producers in Asia and Eastern Africa. Clove oils are currently obtained from dried flower buds, as well as leaves and stems, with the bud oil being most highly valued. Estimated clove oil production for 2016 was 3500–4000 tonnes, with prices ranging from ~$12/kg (clove leaf oil) to ~$80/kg (clove bud oil) (Caiger, 2016).

Eugenol (**4**) has well‐known antioxidant, antimicrobial and anti‐inflammatory properties. It is commonly used as a pesticide and fumigant, as well as to protect foods from micro‐organisms during storage. It also has the property of enhancing skin permeation of various drugs (for a review see Kamatou *et al*., 2012) and is widely used as an analgesic in dentistry (Skinner, 1940; Vassão *et al*., 2010; Weinberg *et al*., 1972).

Isoeugenol (**8**), by contrast, is one of the main volatiles emitted from petunia (*Petunia hybrida*) flowers (Verdonk *et al*., 2003) and is used somewhat as a fragrance/flavour additive in beverages, baked goods, chewing gum and other products such as perfumes and soaps (Badger *et al*., 2002). It also acts as a defence compound *in planta* (Koeduka *et al*., 2008).

It was, therefore, of interest to determine to what extent eugenol (**4**) and isoeugenol (**8**) (or derivatives thereof) could be produced in transgenic poplar in initial (foundational) field trials, as a potential chemical platform.

We describe the genetic engineering of *P. tremula × P. alba* for (i) potentially producing specialty chemicals eugenol (**4**), isoeugenol (**8**) and/or sequestered derivatives thereof, and (ii) establishing what, if any the effects are of such transformations on *P. tremula × P. alba* growth/development and (unexpected) precocious flowering in a field trial environment over a 4‐year time frame. The genes required for the transformations were originally obtained from the creosote bush (*Larrea tridentata*), with proof of principle for eugenol (**4**)/isoeugenol (**8**) production being first demonstrated in *Escherichia coli* (Kim *et al*., 2014). Results obtained are described below.

## Results and Discussion

The biosynthetic pathway to eugenol (**4**)/isoeugenol (**8**) from coniferyl alcohol (**12**) involves an initial acylation step by a substrate versatile monolignol acyl transferase (trivially called coniferyl alcohol acyl transferase (CAAT)) to form, for example, acetate **14** which is converted into eugenol (**4**)/isoeugenol (**8**) via action of either the corresponding allylphenol synthase (APS) or propenylphenol synthase (PPS, Figure 1b) (Kim *et al*., 2014; Koeduka *et al*., 2006; Lewis *et al*., 2015; Vassão *et al*., 2006, 2007). The corresponding genes, namely *LtCAAT*,* LtAPS1* and *LtPPS1*, were cloned from the creosote bush (Kim *et al*., 2014; Lewis *et al*., 2015; Vassão *et al*., 2010) as described in the Supporting Information.

LtAPS1 and LtPPS1 are members of a vascular plant phenylpropanoid oxidoreductase family, which includes pinoresinol–lariciresinol reductase (PLR) in lignan biosynthesis, all of which have very similar biochemical mechanisms (Vassão *et al*., 2010). LtAPS1 and LtPPS1, however, only share moderate homology (49/42% identity and 64/60% similarity, respectively) to PLR from *Forsythia intermedia* (Dinkova‐Kostova *et al*., 1996; Min *et al*., 2003).

### Hybrid poplar transformations

For potential production of allyl/propenylphenols **4** and **8** in transgenic hybrid poplar [as previously carried out for *E. coli* (Kim *et al*., 2014)], *p35S::LtAPS1::T35S* and *p35S::LtPPS1::T35S* gene cassettes were individually excised from their pK2GW7 gateway vector constructs. These were separately fused together with the pK2GW7/*LtCAAT1* vector construct, with constructs harbouring *LtCAAT1*/*LtAPS1* and *LtCAAT1*/*LtPPS1* (Figure S1) individually subjected to an *Agrobacterium* EHA105 transformation procedure (see Costa *et al*., 2013b and Supporting Information).

Transformed *Agrobacterium* EHA105 lines harbouring each pK2GW7 vector construct were then individually incubated with leaves, petioles and internodal stem segments harvested from 50‐day‐old *P. tremula* × *P. alba* (INRA 717‐1B4) for the poplar transformation (see Table S1 and Figure S2).

Quantitative real‐time PCR analyses were next carried out on leaf tissue (see Table S2) using first‐strand cDNA synthesized from total RNA from each plant as a template. For each line, the *LtCAAT1* and *LtAPS1* or *LtPPS1* mRNA expression levels were individually determined, with WT hybrid poplar (*P. tremula *× *P. alba*) and *L. tridentata* employed as comparative controls. (There was, as expected, no detectable expression under the conditions employed with qPCR in the WT lines, when using specific primers for *L. tridentata LtCAAT1, LtAPS1* and *LtPPS1*, hence zero values).

Gene expression levels of *LtCAAT1* and *LtAPS1* in 212 transformed poplar lines growing in the greenhouse ranged from 3 to 5000 and 0.2 to 50 times higher, respectively, as compared to the *L. tridentata* control. Analogously, *LtCAAT1* and *LtPPS1* expression levels were 10–450 and 0.1–18 times higher in the 178 *LtCAAT1::LtPPS1* transgenic lines. Representative transformants (57 APS lines in Figure S3a,b and 46 PPS lines in Figure S4a,b) were selected for subsequent field trials, to cover a broad range of relative gene expression levels.

### Transgenic hybrid poplar field trials

Selected transgenic lines (Figures S3a,b and S4a,b) were analysed for eugenol (**4**), isoeugenol (**8**) and derivatives thereof (**6** and **10**) produced under natural outdoor settings, together with their overall growth/development characteristics relative to WT controls. Growth/development under field trial conditions was particularly important, as one of our other earlier collaborative studies (Voelker *et al*., 2010) had found significant deleterious effects on transgenic poplar growth when 4‐coumarate CoA ligase (4CL) was down‐regulated using comparable approaches as here. That is, some of the 4CL lines in the previous field trials had bushy/stunted growth forms, whereas they were tree‐like under greenhouse conditions. This was not the case though for the poplar producing phenylethanol glucoside (**2**), as the transformants grew normally in field trials (Costa *et al*., 2013b).

Transgenic poplar parent plants above were delivered for field trial evaluation in September and October of 2011, with each parent being *ca*. 30–60 cm tall. These were grown in 1‐gallon containers in greenhouses, under 16‐h light and 24.5 °C day temperature and 20 °C night temperature conditions. Fertilizer (Wilgro, Wilbur‐Ellis, 20:20:20) was supplied for every watering at 600‐μs conductivity (incoming H_2_O 208 μs), with parents flushed every 10 days for 3 days with only incoming H_2_O to reduce pot salt load. The first cuttings from the parents were placed in rooting trays in December 2011, then transplanted in Beaver Plastics 45 cavity trays in February 2012 and further grown in the greenhouse to the end of March 2012. Thereafter, transplanted cuttings with a 3 to 4‐mm caliper above the original cutting stem were moved to cold frames and grown until planting specification sizes of 45–60 cm in height were obtained. These thus provided the biological replicates needed for the field trial described below.

The field trial with these lines was planted over 4 days in June (June 4th to 8th) 2012 (see Experimental procedures for details). The complete trial (together with other transgenic lines) was planted at 1.52 × 1.52 m spacing to fit a total of 5019 trial trees inside the area allocated by the USDA APHIS permit. There were 20 replicates, generated as described in the paragraph above, for each WT and transgenic line, these being placed in a statistically random design.

In 2012, stem height measurements were made, whereas in 2013 and 2014 (see Figure S5), plant volume production was estimated by measuring stem heights and diameters. There was no obvious massive deleterious effect on height measurements through increasing (introduced) gene expression levels, even though some variations in growth were noted between different lines.

The trial design had very good statistical power, where 20 replicates provided 93% power at a 95% confidence limit; that is, designed so that the results supported the null hypothesis that there was a significant difference of 30 cm of growth between clones. This is a standard clonal forestry trial layout design, which would generally be used to select for the best growing clones across a trial site. The variation observed, however, was not in fact due to the site but instead competition between plant lines.

The aim of the trial here was to find the largest tree with the highest desired metabolite of interest level—in contrast to a production setting, which would select for the biggest tree with most wood product. With these trees, some lines grew slower. However, as the trial closes canopy (about year 2.5), then shorter trees cannot compete well for light and because of that also water and nutrients. To try and compete, they grow thinner and taller to try to capture some extra canopy growing space. Consequently, some cannot keep up and their diameters become more and more variable across the trial, an expected result in such trials. The key here, however, was in trying to select for the biggest volume tree with highest levels of metabolites of interest, with the smaller trees becoming more variable as they attempt to compete and are outgrown.

Field trial evaluations from 2012 to July 2016 were carried out under USDA APHIS approval, which allows for growth/development periods of 3–5 years, provided no flowering/pollination occurs with either transgenic lines or any adjacent/nearby WT poplars. This current requirement is to ensure that there is no ‘escape’ of the transgenic lines.

Unexpectedly, during Spring 2016 (4th growing season), 114 transgenic trees from 36 of the lines carrying the *LtCAAT1/LtAPS1* genes (Figure S3c) and 23 transgenic trees from 16 of the lines carrying the *LtCAAT1/LtPPS1* genes (Figure S4c) flowered precociously and were felled according to USDA APHIS requirements. (The USDA APHIS permit does not allow flowering, and the trees were felled as soon as catkins were noted.) Flowering was heavier either in the outer edges of the trial or where LtAPS and LtPPS lines were in close proximity to each other in the inner areas. However, as for stem height and diameter data, there was no direct correlation noted between expression level of introduced genes and trees that flowered (Figures S3 and S4). The reason(s) for precocious flowering is (are) not known at this time and could not have been anticipated. Precocious flowering has, however, been very recently reported with transformed *Eucalyptus* lines, although in that case, this was expected as they were specifically engineered to shorten flowering time (Klocko *et al*., 2016b). Precocious flowering has two possible ramifications for commercialization: (i) plant lines are coppiced within 3 years or (ii) emerging gene editing procedures are further developed to prevent flowering onset (Klocko *et al*., 2016a).

### Metabolomics

In the field trial, eleven of each of the different LtAPS and LtPPS transgenic poplar plant lines were selected for both targeted and nontargeted metabolomics analyses, with the remainder assessed for growth/development parameters. As indicated earlier, each line had 20 replicates in the trial randomly configured. Selected lines for metabolomics analyses were chosen to cover a broad range of *LtCAAT*,* LtAPS* and *LtPPS* gene expression levels, with six of the 20 replicates being analysed. In particular, it was of interest to establish what, if any, correlations could be made between gene expression and bioproduct accumulation level.

#### Targeted metabolomics analyses

Leaf and branch tissues from selected lines were individually harvested (August 2014, after *ca*. 2 years growth), extracted and analysed (see Experimental procedures). Two complementary approaches were utilized: (i) GCMS analysis of *t*‐butyl methyl ether (containing 0.5 mm benzyl methyl ether internal standard (IS)) extracts of fresh tissue to determine amounts of ‘free’ eugenol (**4**), chavicol (**3**), isoeugenol (**8**) or *p*‐anol (**7**) present in foliage or branch tissues, and (ii) LC/MS analyses of aqueous methanolic extracts of freeze‐dried tissue to establish amounts, if any, of sequestered allyl/propenylphenols, such as the glucosides (**5**,** 6**,** 9** and **10**) or as other derivatives. [In this context, glycosylation is a very well‐known mechanism *in planta* to sequester phenolic and other alcohol functionality storage products, for example phenylethanol glucoside (**2**) in transgenic poplars (Costa *et al*., 2013b).]

**Figure 2:**
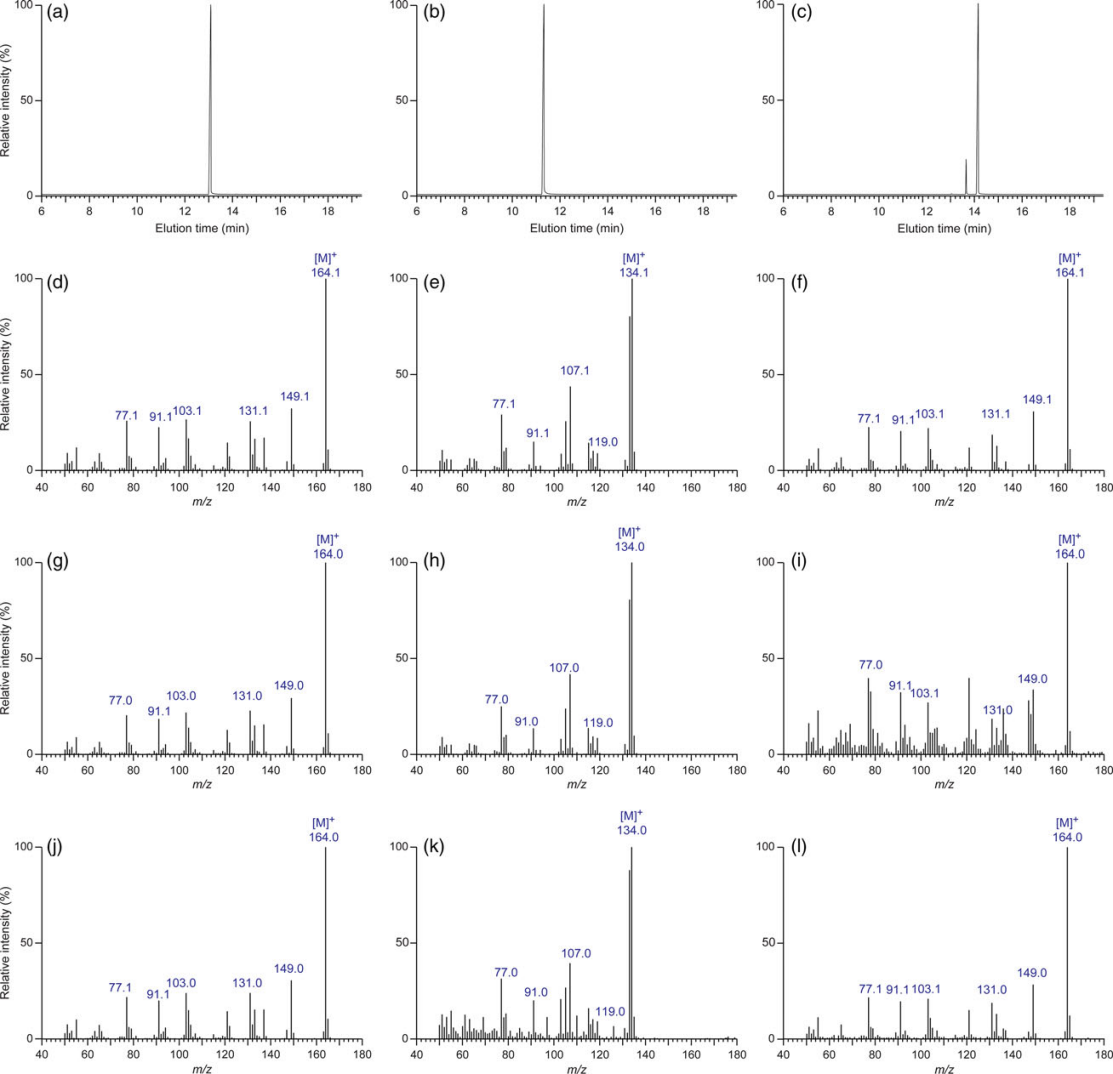
Eugenol (**4**)‐, chavicol (**3**)‐ and isoeugenol (**8**)‐extracted ion chromatograms and their mass spectra. Extracted ion chromatograms (a–c) of eugenol (**4**), chavicol (**3**) and isoeugenol (**8**) standards correlated with elution times, and their respective mass spectra (d–f). Mass spectra of eugenol (**4**), chavicol (**3**) and isoeugenol (**8**) in LtCAAT1/LtAPS1 (g–i) and LtCAAT1/LtPPS1 (j–l) transgenic hybrid poplars, respectively.

For assessments of eugenol (**4**), chavicol (**3**), isoeugenol (**8**) and *p*‐anol (**7**) accumulating in the various transformed poplar lines, relative to WT, analyses (including identification and quantification) were on a fresh weight basis. In this regard, while eugenol (**4**), chavicol (**3**) and isoeugenol (**8**) were commercially available as standards, *p*‐anol (**7**) was synthesized from *p*‐hydroxybenzaldehyde via reaction with ethyl triphenyl phosphonium iodide in the presence of *n*‐BuLi in *ca*. 86% yield (Kim *et al*., 2014). Conditions for their chromatographic resolution, and establishing their mass spectroscopic fragmentation patterns, were developed, as well as for GC/MS calibration and recovery. Only data for eugenol (**4**), chavicol (**3**) and isoeugenol (**8**) standards are shown in Figures 2a–f, as *p*‐anol (**7**) was not detected in the plant sample analysed.

For plant analyses (harvesting, storage, extraction and recovery), authentic standard calibrations and spiking of tissues with standards individually (see Supporting Information) were carried out, with results normalized by use of the benzyl methyl ether IS, to account for recovery losses, etc. In WT leaf tissues, eugenol (**4**), chavicol (**3**) and isoeugenol (**8**) were in trace amounts (near detection limits), these being ≤0.0006, 0.0001, and 0.00005% fresh weight, respectively, with *p*‐anol (**7**) not detected under the conditions employed. By contrast, in the LtAPS lines, amounts of eugenol (**4**), chavicol (**3**) and isoeugenol (**8**) in leaf tissue increased somewhat in amounts of up to 0.01, 0.0007 and 0.00009% fresh weight, that is 167, 70 and 1.8 times higher, respectively (data not shown). For LtPPS lines, amounts of eugenol (**4**) in leaf tissue increased to somewhat similar levels (up to 0.009% fresh weight), with chavicol (**3**) and isoeugenol (**8**) being present in amounts of *ca*. 0.0005% and 0.0004%, respectively [All metabolites were identified based on same retention times and mass spectral fragmentation patterns (Figures 2g–l) as compared to authentic standards (Figures 2a–2f)]. Formation of chavicol (**3**) was not unexpected, given the known substrate versatility of the cloned enzymes, which can utilize other monolignols such as *p‐*coumaryl alcohol (**11**) (Kim *et al*., 2014).

It was next instructive to ascertain whether allyl/propenylphenols **5**,** 6**,** 9**,** 10** were accumulating in sequestered form in leaf tissues, such as noted for phenylethanol glucoside (**2**) (Costa *et al*., 2013b). Thus, eugenol glucoside (**6**), isoeugenol glucoside (**10**), chavicol glucoside (**5**) and *p*‐anol glucoside (**9**) were individually synthesized (see Supporting Information), with general synthetic protocols employed being described using eugenol glucoside (**6**) as an example. That is, α‐acetobromoglucose was reacted with sodium eugenolate (obtained from eugenol (**4**) and sodium ethoxide) to generate eugenol‐β‐glucoside (**6**). After crystallization, its β‐configuration was determined by analysis of its one‐ and two‐dimensional homo‐ and heteronuclear ^1^H spectra in dimethylsulphoxide‐d6 solutions, with the β‐glucopyranosyl linkage deduced from the higher coupling constant (*J* = 7.32 Hz) and lower δ (4.78 ppm) value of the anomeric proton (Mastelic *et al*., 2004).

**Figure 3:**
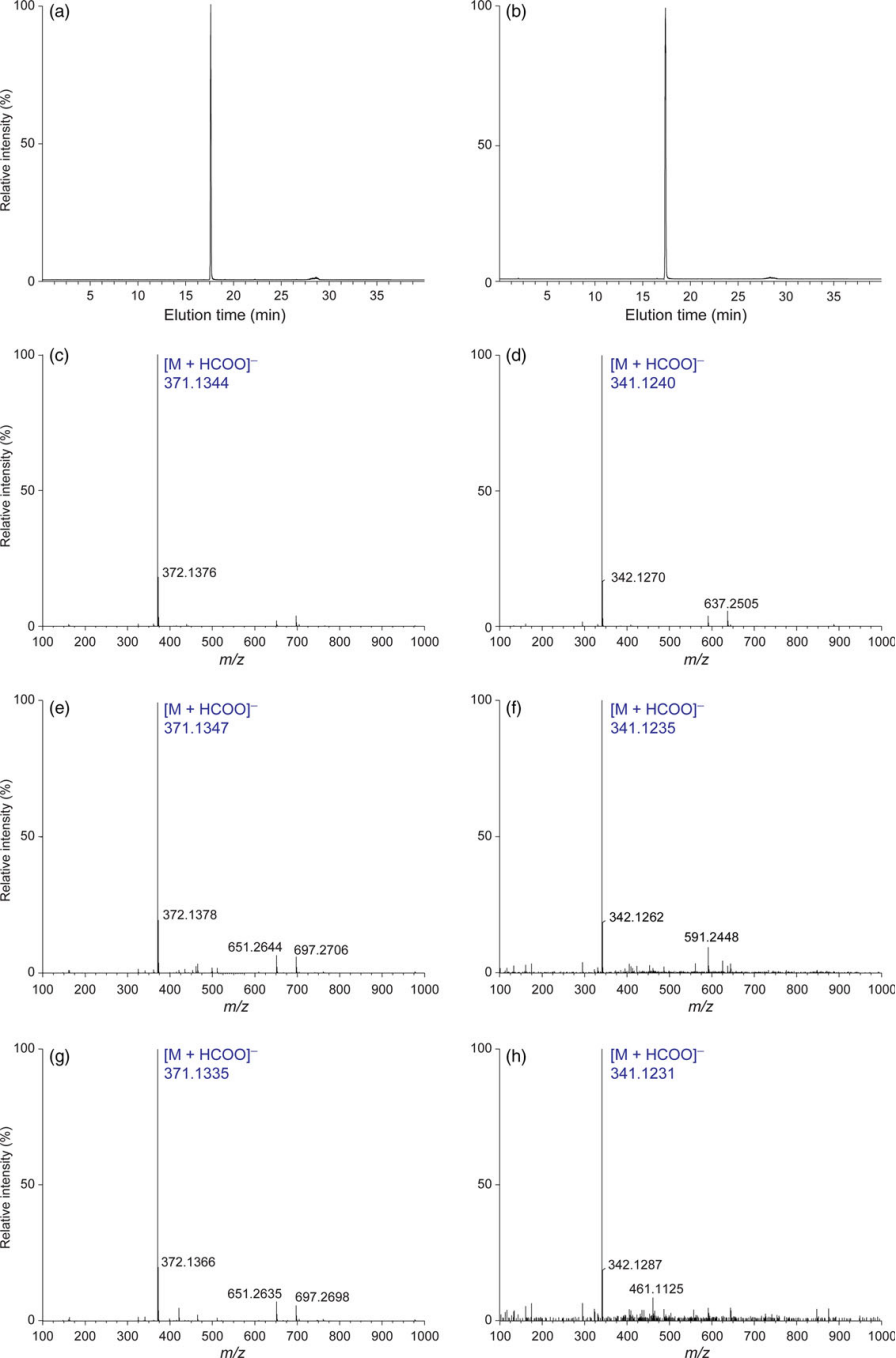
Eugenol glucoside (**6**)‐ and chavicol glucoside (**5**)‐extracted ion chromatograms and their [M + HCOO]^−^ molecular ion mass spectra. Extracted ion chromatograms of (a) eugenol glucoside (**6**) and (b) chavicol glucoside (**5**) standards correlated with elution times, and their single ion patterns (c and d), respectively. Single ion patterns of eugenol glucoside (**6**) and chavicol glucoside (**5**) in LtCAAT1/LtAPS1 (e, f) and LtCAAT1/LtPPS1 (g, h) transgenic hybrid poplars, respectively.

Similarly, glucosides **5**,** 9** and **10** were synthesized, and all four glucosides were used for calibration curve standardization and normalization to the naringenin IS. Both eugenol glucoside (**6**) and chavicol glucoside (**5**) were detected as [M + HCOO]^−^ ions. Conditions for their separation and calibration of extraction protocols were then developed (Figures 3a–d) in the negative ion mode, following which analyses of aqueous methanolic extracts of WT, LtAPS and LtPPS leaf tissues were carried out. For WT leaf tissue, allyl/propenylphenol glucosides **5**,** 6**,** 9** and **10** were not unambiguously detected/identified, as all were below detection limits with the methodologies employed (data not shown).

By contrast, with LtAPS lines, eugenol glucoside (**6**) and chavicol glucoside (**5**) were unambiguously identified in the leaf tissues, based on elution time, UV and mass spectral fragmentation patterns (see Figures 3e,f), whereas neither isoeugenol glucoside (**10**) nor *p*‐anol glucoside (**9**) were detected. Quantification of glucoside **6** ranged from 0.25 to 0.35% dry weight relative to both the standard glucoside **6** calibration and the naringenin IS recovery (see Supporting Information). For the eugenol glucoside (**6**) standard, the ion at *m/z* 371.1344 corresponded to [M + HCOO]^−^ (Figure 3c) with the same ion observed in the LtAPS line (Figure 3e). The amounts of chavicol glucoside (**5**) at *m/z* 341.1235 (Figure 3f) ranged from 0.07 to 0.1% dry weight.

With LtPPS leaf tissues, both eugenol glucoside (**6**) and chavicol glucoside (**5**) were present (see Figures 3g,h), but considerably lower in amounts ranging from 0.04 to 0.09 and 0.006 to 0.015% dry weight, respectively. Eugenol glucoside (**6**) had a *m/z* 371.1335 [M + HCOO]^−^ ion, whereas chavicol glucoside (**5**) had a *m/z* 341.1231 [M + HCOO]^−^ ion. Expected products, isoeugenol glucoside (**10**) and *p*‐anol glucoside (**9**) were not detected.

**Figure 4:**
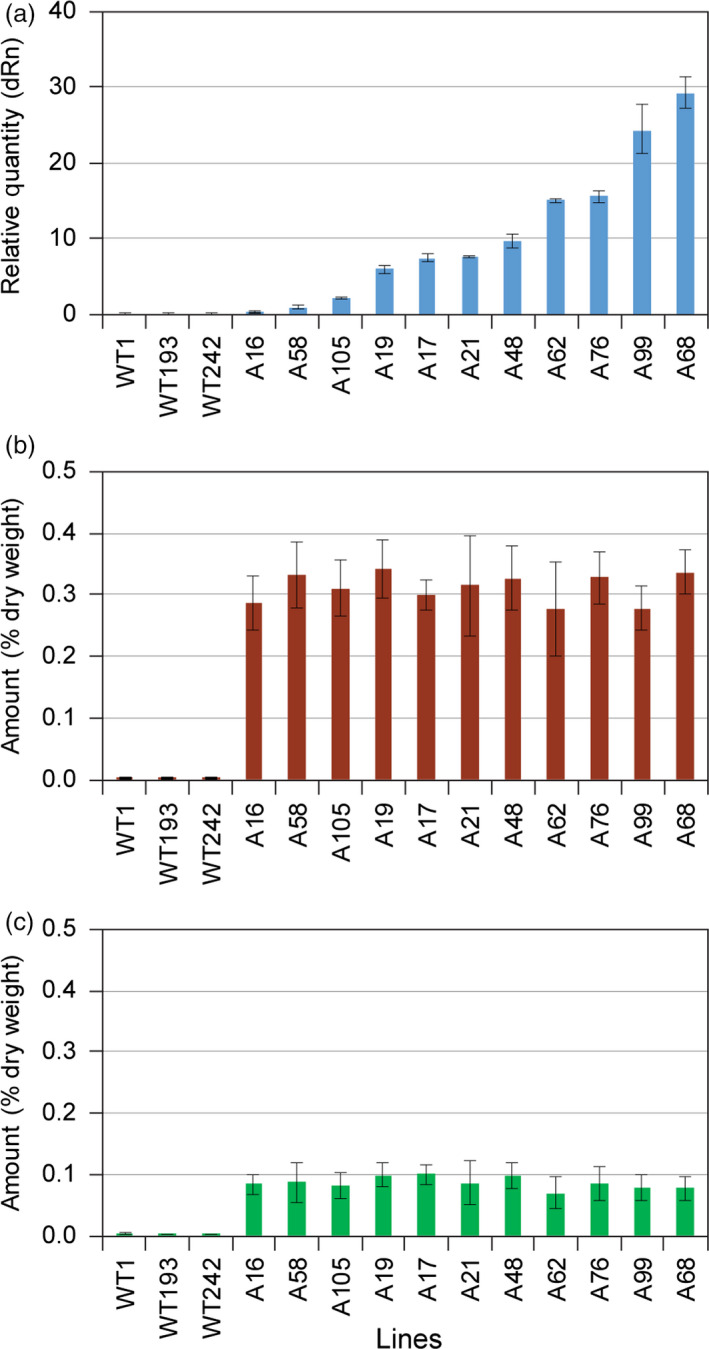
Correlation of engineered pathway expression levels with eugenol glucoside (**6**) and chavicol glucoside (**5**) amounts in transformed hybrid poplar. (a) Expression levels of *LtAPS1* in hybrid poplar transformed with the *p35S::LtCAAT1::T35S::p35S::LtAPS1::T35S* construct. Accumulation levels of (b) eugenol glucoside (**6**) and (c) chavicol glucoside (**5**) in leaves of WT and transgenic hybrid poplars harvested in August 2014.

**Figure 5:**
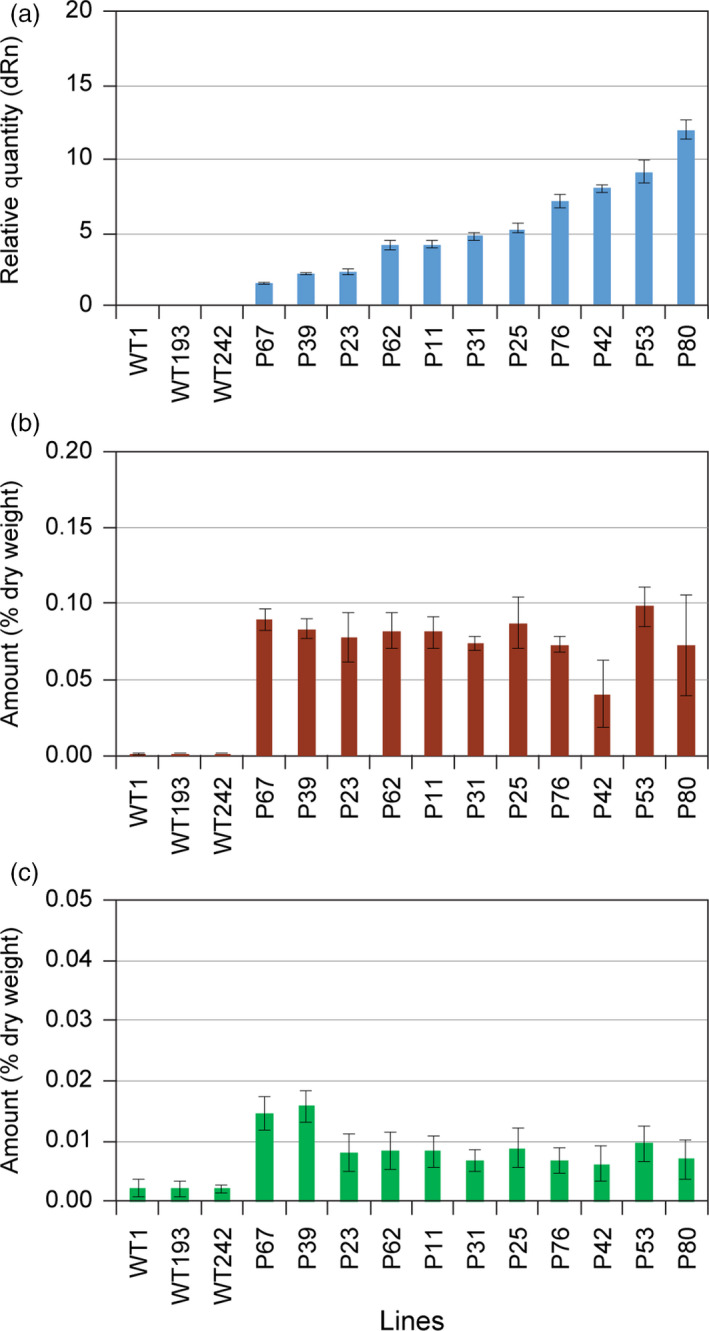
Correlation of engineered pathway expression levels with eugenol glucoside (**6**) and chavicol glucoside (**5**) amounts in transformed hybrid poplar. (a) Expression levels of *LtPPS1* in hybrid poplar transformed with the *p35S::LtCAAT1::T35S::p35S::LtPPS1::T35S* construct. Accumulation levels of (b) eugenol glucoside (**6**) and (c) chavicol glucoside (**5**) in leaves of WT and transgenic hybrid poplars harvested in August 2014.

It was next instructive to compare the 11 selected LtAPS and LtPPS lines as regards their differential levels of gene expression (Figures 4a and 5a) and accumulation levels of eugenol glucoside (**6**) (Figures 4b and 5b) and chavicol glucoside (**5**) (Figures 4c and 5c), respectively. In the LtAPS lines, while gene expression levels ranged from 1 to 30, there was essentially no effect on eugenol glucoside (**6**) accumulation levels, with these reaching a maximum plateau value even at the lowest gene expression level. The same trend was noted for chavicol glucoside (**5**) levels, except they were about one‐third of eugenol glucoside (**6**) amounts (Costa *et al*., 2013a; Kim *et al*., 2013).

Provisionally, these data suggest that there was limited substrate monomer supply, or that some other form of metabolic block (including pathway inhibition) was operative, that is, as metabolite levels did not increase with increasing level of gene expression. This result thus contrasted with our findings (Costa *et al*., 2013b) of much higher accumulation levels of phenylethanol glucoside (**2**), these being *ca*. >10‐fold higher than for the combined amounts of compounds **5** and **6**, respectively, even though they were all derived from Phe. However, phenylethanol glucoside (**2**) is directly produced from available Phe, whereas eugenol (**6**) and chavicol (**5**) glucosides are much further downstream in terms of metabolic pathway placement. Indeed, in other studies with transgenic *Arabidopsis*, a three‐step pathway from tyrosine to dhurrin (Kristensen *et al*., 2005; Morant *et al*., 2007) gave somewhat similar levels of metabolite accumulation to that observed for phenylethanol glucoside (**2**) (Costa *et al*., 2013b). An assumption here, though, is also that the accumulation levels of **5** and **6** represent terminal storage products.

Analogously, comparison of LtPPS gene expression levels was also very informative, in terms of accumulation of eugenol glucoside (**6**), chavicol glucoside (**5**) and *p*‐anol (**9**) glucoside. Similar trends were noted, in terms of metabolite levels, except that amounts of (**6**) were much lower (by a *ca*. 3‐fold reduction), whereas those of (**5**) remained about the same, relative to the LtAPS observation. This was unexpected, as in *E. coli*, LtPPS produces isoeugenol (**8**), but not eugenol (**4**) (Kim et al., 2014). Interestingly, very small amounts of one of the possible products, *p‐*anol glucoside (**9**), were observed, but its levels did not increase with increasing gene expression level. To account for the absence of **8**, perhaps its fully conjugated (more reactive) nature enabled it to undergo phenoxy radical coupling more readily *in situ* (i.e. thus reflecting a potential increased susceptibility of the more highly conjugated (**8**) as compared to eugenol (**4**)).

**Figure 6:**
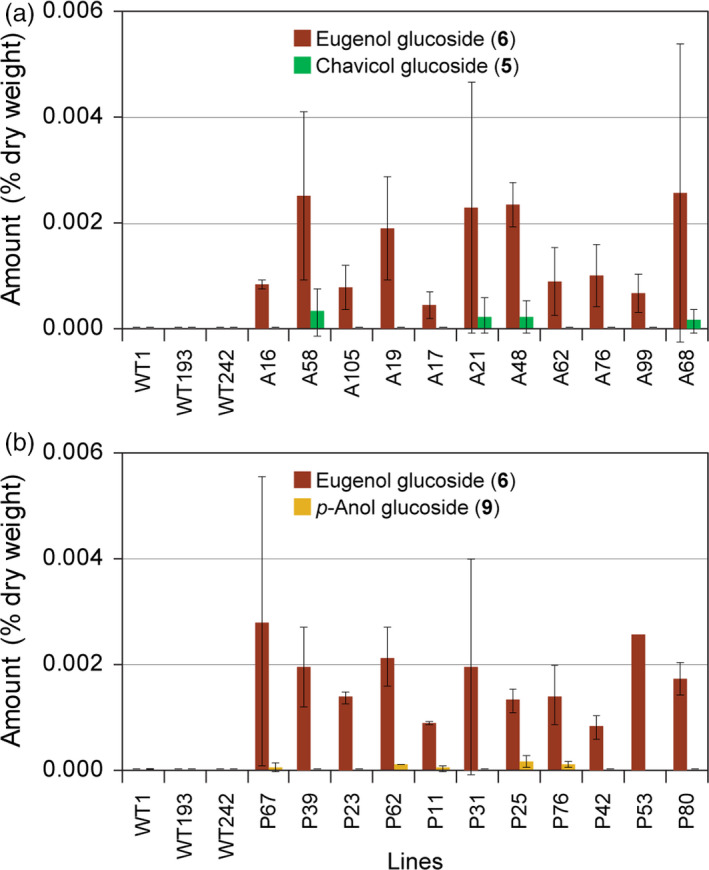
Accumulation levels of eugenol glucoside (**6**), chavicol glucoside (**5**) and *p‐*anol glucoside (**9**) in branches of WT and transgenic hybrid poplar expressing *LtAPS* (a) and *LtPPS* (b).

The aqueous methanol extracts of branch tissues from WT, LtAPS and LtPPS lines were also analysed to determine whether sequestered products **5**,** 6**,** 9** and **10** were present. None were, however, detected in WT branches, although both LtAPS and LtPPS lines contained eugenol glucoside (**6**) at very low but similar levels, with amounts ranging from 0.00008 to 0.003% dry weight (Figures 6a,b). By contrast, chavicol glucoside (**5**) was only detected in LtAPS lines in very low amounts of *ca*. 0.0002% dry weight (Figure 6a), whereas isoeugenol glucoside (**10**) was again not detected; *p*‐anol glucoside (**9**) was potentially detected in trace amounts (*ca*. 0.00004% dry weight) in the LtPPS lines (Figure 6b).

#### Nontargeted metabolomics analyses

In addition to the above metabolic products, an untargeted metabolomic analysis of the UPLC‐qTOF data was performed to establish whether other unforeseen metabolic effects occurred, with a particular focus on phenylpropanoid and/or upstream shikimate–chorismate pathway metabolite/metabolic pools. Negative ion mode data were thus processed using XCMS and CAMERA R packages for the untargeted metabolomic analyses, as this mode works quite well for phenolic/aromatic ring compounds in *Populus* species. Such metabolites are mainly derived from phenylpropanoid and/or shikimate–chorismate pathways. Indeed, several peaks in HPLC chromatograms of polar extracts have been putatively annotated as flavonoid‐ and salicylate‐derived compounds (Morreel *et al*., 2006). However, data for unambiguous identification of these metabolites was lacking. In any event, our approach led to detection of 1102 features (i.e. unique *m/z* vs. RT signals integrated during XCMS processing) in 24 plant lines, of which 280 were significantly different in comparison of transgenic and control plants (*P* ≤ 0.05). [This approach, however, depends on careful analysis of the corresponding pseudo MS2 spectra extrapolated by the CAMERA tool, that is based on chromatographic behaviour of the different features that were grouped as belonging to the same metabolite.]

In addition to eugenol glucoside (**6**) and chavicol glucoside (**5**), another eugenol (**4**) derivative, reported as being similar (Koeduka *et al*., 2013; Tikunov *et al*., 2013) but not identical to eugenol‐xylosyl‐glucoside (Straubinger et al., 1994) was detected (Tables S3 and S4). While this metabolite was not fully identified, its presence underscores the importance and diversity of metabolism that can occur in engineered pathways, thereby contributing to increasing the sink and productivity in such plant biofactories.

There were also some 14 other metabolites whose levels showed differences (up to <1.67‐fold) between transgenic and WT lines. Supplemental Tables S3 and S4 and Figure S6 summarize the detection of these molecular species, their fold changes between WT and transgenic lines, and their tentative structural types. These were not explored further in this study given the relatively small (fold) changes noted in their amounts. The transgenic plant lines also had reduced levels of a syringic acid glucose derivative relative to WT, that is by *ca*. 0.83‐ and 0.71‐fold reductions (p values of 0.1431, 0.0046, respectively; Tables S3 and S4). However, whether this metabolite was phenolically linked (Wolfram *et al*., 2010) or ester‐linked (Yue *et al*., 1994) was not further investigated.

### Cell wall analyses

Analyses of polymeric carbohydrates (cellulose, hemicelluloses), as well as lignin and matrix sugars, were also carried out for branch samples (see Figures S7–S10); differences noted, however, were provisionally considered being due to variability in branch growth/development on sampling.

### Lessons learned and future directions

This foundational study assessed whether introduction of biochemical pathways into poplar to potentially produce eugenol (**4**), chavicol (**3**), *p*‐anol (**7**) and/or isoeugenol (**8**), as well as corresponding sequestered storage products **5**,** 6**,** 9** and **10**, could be achieved. This approach differed from an earlier successful undertaking to produce phenylethanol (**1**), via biotechnologically introducing a two‐enzyme step process from Phe in poplar foliage tissue (Costa *et al*., 2013b). This was because the study here required availability of much farther downstream phenylpropanoid pathway substrates, namely the monolignols coniferyl (**12**) and *p*‐coumaryl (**11**) alcohols, respectively. As poplar foliage produces many Phe‐derived phenylpropanoid (acetate) products, such as the aforementioned flavonoids and other pathway‐related metabolites, it was by no means certain that carbon flux to monolignol substrates vs. other phenylpropanoid (acetate) pathway metabolites would be favoured with these manipulations.

The data obtained established that the various poplar lines generated were not only transformed with the biochemical pathways of interest, but that eugenol glucoside (**6**) and chavicol glucoside (**5**) accumulated in foliage tissue in ~0.35 and ~0.1% dry weight, as well as traces of corresponding aglycones. By contrast, they were only traces of these metabolites in branch tissues.

On the other hand, and unlike the earlier *E. coli* study (Kim *et al*., 2014), neither isoeugenol (**8**) nor *p*‐anol (**7**) accumulated, perhaps due to either further downstream metabolism of these metabolites or the enzyme kinetics being such that they could not favourably compete for monolignols **12** and **11** in the foliage tissues.

While various LtAPS and LtPPS lines accumulated some of the desired products in their foliage tissues, their accumulation levels reached a plateau even at lowest gene expression levels achieved. Their formation also had no obvious massive effect on plant productivity (volume and diameter), in contrast to previous field trial analyses (Voelker *et al*., 2010). As increased gene expression in the various lines did not result in increased accumulation levels of the targeted bioproducts, this raises the exciting possibility of, in future, significantly increasing the metabolite levels through further iterations.

The eugenol glucoside (**6**) bioproducts production, harvesting and processing to this point in poplar foliage tissue allow for considering potential entry into economically displacing the current annual worldwide production of around 4000 tonnes of natural clove eugenol (**4**) (with pricing of *ca*. $12‐80/kg, or $48‐320M revenue annually). Clove production in 2013 in Tanzania, for example, was about 1.14 tonnes/ha, and whose clove oil content can range between 2 and 2.5% (22.8 ‐28.5 kg/ha) (FAOSTAT; World Agriculture). This, in turn, gives maximum eugenol (**4**) amounts of *ca*.18.2–22.4 kg/ha at an estimated 80% eugenol (**4**).

In this regard, full‐size trees of commercial poplar (*Populus deltoides × P. trichocarpa)* species can produce *ca*. 2.2 tonnes/ha of leaf biomass which could give approx. 8 kg/ha of eugenol glucoside (**6**), or *ca*. 4 kg/ha eugenol (**4**). Thus, to meet the 4000 tonnes current production levels of eugenol (**4**) from clove, some 800 000–1 M ha of land would be required. Increasing levels of their amounts by up to *ca*. 40‐fold or so would reduce the land need down to 20–25 000 ha, and potentially produce value‐added bioproducts (in addition to wood and pulp) of $48–320 M additional revenue annually. Currently, clove production worldwide uses in excess of 400 000 ha (World Agriculture).

Accordingly, in future, studies will employ numerous ‘omics’, systems biology, synthetic biology and metabolic modelling approaches to next identify and overcome metabolic blocks/restricted carbon flux to the desired products in both foliage and stems. Furthermore, while some LtAPS and LtPPS lines displayed precocious flowering after a 4‐year growth period, there are biotechnological approaches available (e.g. gene editing) to produce sterile plant lines which would potentially prevent this unexpected finding from occurring.

## Experimental procedures

### Field trial

For isolation/cloning of *LtCAAT1‐*,* LtAPS1‐* and *LtPPS1*‐coding genes and vector construction for heterologous expression, hybrid poplar transformation, total RNA isolation and quantitative real‐time PCR for selection of transgenic poplar, see Supporting Information.

The field trial, planted in June 2012, consisted of a total of 5019 poplar trees (*P. trichocarpa* and *P. tremula *×* P. alba*) derived from 245 mixed transgenic lines (4883 trees) and 136 WT (20 *P. trichocarpa* and 116 *P. tremula* × *P. alba*). This included the 57 *P. tremula *×* P. alba* lines (1126 trees) transformed with the LtCAAT‐LtAPS vector and the 46 *P. tremula *×* P. alba* lines (905 trees) transformed with the LtCAAT‐LtPPS vector, as well as the 116 WT *P. tremula *×* P. alba* as controls. The layout was a completely randomized single‐tree plot design with 20 biological replications (Jayaraman, 1999). The trial was planted as a 1.52 m × 1.52 m square lattice in June 2012. Irrigation was by means of drippers (2 L/h) installed in 17‐mm LDPE piping (Netafim, Fresno, CA, USA) through Triton X irrigation, with watering set for 1 h twice a day so that each tree received approx. 4 L H_2_O per day. After the initial settling period, fertilizer (Wil‐Sol^®^ Pro‐Balance 20‐20‐20; Wilbur Ellis, Aurora, CO, USA) was supplied at an 1:120 dilution ratio via a proportional dosing pump (D45RE15‐20 GPM; Dosatron, Clearwater, FL, USA) as follows: 14.3, 16.1, and 12.4 g of concentrate per tree was applied in years 1, 2 and 3, respectively. Once the trees began to go dormant, a chemical hardener fertilizer (Wil‐Sol^®^ Pro‐Finisher 4‐25‐35; Wilbur Ellis) was applied at the same dilution giving 4.4, 5.6 and 7.4 g of concentrate per tree in years 1, 2 and 3, respectively. Watering ceased after completion of the third growing season.

Tree heights and diameters were measured, tree heights only for the first year (2012) and then heights and diameters yearly (e.g. 2013 and 2014). Heights were measured for the first 3 years using a height pole, and then, a Nikon Laser Forestry Pro Rangefinder 8381 was used to determine heights using three‐point measurement. Diameters were measured with a 0‐25 cm tree caliper rotated around the stem at the standard nominal breast height (1.4 m)—diameter at breast height (DBH).

### Plant harvesting

The bottom branches and their leaves were individually collected from selected lines (replicates 10–15) as per USDA APHIS required protocols: Branches, cut into 10‐cm sections, and leaves from each tree were separately flash‐frozen in liquid N_2_, kept on dry ice until reaching destination, and stored at −80 °C until sample processing.

### Metabolite extractions

For allyl/propenylphenol determinations, frozen leaf tissues (0.2–0.5 g) were individually ground to a fine powder using a TissueLyser II (Qiagen, Germantown, MD, USA; 30 s at a frequency of 30 Hz) equipped with one 5‐mm steel ball, with resulting ground tissues stored at −80 °C until needed. Lower branch tissues, by comparison, were first chopped into small pieces, ball‐milled into a fine powder for 6 h and sieved (600 μm).

To each tissue sample type, 300–500 mg was added *tert*‐butyl methyl ether (2 μL per mg tissue) containing 0.5 mm benzyl methyl ether as internal standard (IS). Each tissue was vortexed for a few seconds, sonicated in cold H_2_O for 15 min, vortexed again and centrifuged (20 000 *g*, 15 min, 4 °C). Each supernatant was subjected to GC‐MS analysis.

For allyl/propenylphenol glucoside determinations, freeze‐dried leaves and branch samples from each line were prepared as above. Each sample was individually extracted with MeOH–H_2_O (70:30, v/v) containing 0.1 mm naringenin IS at a ratio of 1 mL to 50 mg of tissue. After adding extraction solvent, each solution was vortexed for a few seconds, sonicated in cold H_2_O for 15 min, vortexed again and centrifuged (20 000 *g*, 15 min, 4 °C). Each supernatant was transferred individually to UPLC vials and subjected to UPLC‐QTOF‐MS analyses.

### GC‐MS analyses

These were carried out on a HP 6890 Series GC System equipped with a HP 5973 MS detector (EI mode, 70 eV). Separations of metabolites were achieved using a RESTEK‐5Sil‐MS (30 m × 250 mm × 0.25 mm) column, with column conditions as follows: 40 °C maintained for 2 min, then 40–150 °C at 10 °C/min and 150–250 °C at 20 °C/min with a final holding time of 2 min; total run time was 20 min, with a detector temperature of 250 °C. Amounts of eugenol (**4**)/isoeugenol (**8**) and chavicol (**3**)/*p*‐anol (**7**) were estimated based on *m/z* 164 and 134 extracted ion traces, respectively. Peak areas were normalized to the benzyl methyl ether IS peak area and quantified using calibration with authentic standards.

### UPLC‐QTOF‐MS analyses

UPLC‐QTOF‐MS analyses employed a Waters Acquity™ Ultra Performance LC system (Waters, Milford, MA) equipped with a photodiode array (PDA) eλ detector (Waters) coupled to a Xevo™ G2 QTof mass spectrometer (Waters MS Technologies, Manchester, UK) using MassLynx (V4.1) software. Separations of metabolites used a ACQUITY UPLC^®^ BEH C_18_ column (Waters, 2.1 × 150 mm, 1.7 μm particle size), with a linear gradient for separation: 100% A (0.1% HCO_2_H in H_2_O) over 0.5 min, 0–45% B (0.1% HCO_2_H in CH_3_CN) over 25.0 min, 45–100% B over 1.0 min, held at 100% B for a further 2.0 min and then re‐equilibrated at 100% A for 11.5 min. Flow rate was 0.2 mL/min, with column and sample temperatures kept at 25 and 5 °C, respectively. Injection volumes were 2 μL for LC‐MS analysis and 4 μL for LC‐MS/MS, with UV–visible spectra recorded between 200 and 500 nm (1.2 nm resolution). An electrospray ionization (ESI) source was used to detect masses of eluted compounds (*m/z* range: 50–1000 Da) and Ar was the collision gas. Detection settings were as follows: Negative ion mode: capillary voltage at 2.0 kV, cone voltage at 30 eV, collision energy at 6 eV and at 27 eV. Positive ion mode: capillary voltage at 3.0 kV, cone voltage at 30 eV, collision energy at 6 eV and at 18 eV. For LC‐MS/MS analyses, 5–50, 30 and 30–60 eV collision energies were used. Sodium formate (5 mm in 2‐propanol‐H_2_O, 90:10, v/v) was used for calibrating the mass spectrometer, with leucine enkephalin (2 ng/μL in CH_3_CN − H_2_O + 0.1% HCO_2_H, 50:50, v/v) employed as lock mass. For extraction protocol standardization and calibration, see Supporting Information.

### Metabolomics data processing and normalization

After UPLC‐QTOF‐MS analysis, chromatograms were reviewed using MassLynx software (Waters) with obvious outliers excluded. To conduct subsequent data processing, RAW data files were converted to NetCDF using MassLynx's Databridge software. NetCDF files were processed in RStudio (V 0.9.332) using the package XCMS (Smith *et al*., 2006) for feature (unique mass/retention time signals, MxxxTxxx) detection and retention time correction, followed by the CAMERA package (Kuhl *et al*., 2012) for features grouping and partial annotation (https://bioconductor.org/packages/release/bioc/html/CAMERA.html). XCMS‐based feature detection and integration were performed using the Centwave method (Tautenhahn *et al*., 2008) with the following parameters: prefilter = c(0,0), snthr = 2, ppm = 15, peakwidth = c(5,20), and nSlaves = 4. Retention time correction was performed twice via the obiwarp method as follows: bw = 2, minfrac = 0.5, mzwid = 0.015 and plottype = c (‘deviation’). The CAMERA package was also used as follows: perfwhm = 0.6 and groupCorr, findIsotopes and findAdducts with polarity = ‘negative’.

Pairwise comparison in XCMS was conducted after setting the two groups, one including all WT samples and the other samples from all transformed lines (both LtAPS and LtPPS). After initial XCMS and CAMERA processing, TSV and CSV files generated were combined and further analysed manually. Feature lists were normalized to the naringenin IS (integrated in XCMS results as feature M271T1315; retention time (RT) 21.92; *m/z* 271.0607, calculated 271.0606 for C_15_H_11_O_5_) for statistical analysis. Comparisons between WT and transgenic lines (either from LtAPS or LtPPS plants) were performed separately for selection of statistically significant features, including being subjected to provisional annotation efforts.

### Nontargeted metabolite provisional annotation

Following computational processing, information obtained was used for manual peak identification of known and putatively annotated poplar metabolites. Targeted known metabolites were identified by comparison with retention time, UV spectrum and MS/MS fragmentation of authentic standards. Nontargeted metabolites were putatively annotated as follows: (i) molecular formulae calculated from accurate mass and isotopic pattern recognition were used for screening Metlin (https://metlin.scripps.edu/index.php) and SciFinder Scholar metabolite databases (SciFinder Scholar™ 2015), as well as other sources including KEGG (http://www.genome.jp/kegg/pathway.html) and Plant Metabolic Network (PMN, http://www.plantcyc.org/) databases. (ii) MS/MS fragmentations of nontargeted metabolites were compared with candidate molecules found in databases [ReSpect for Phytochemicals (http://spectra.psc.riken.jp/) and MassBank (http://www.massbank.jp/)] and verified with literature reports, especially for metabolites (known and/or putative) previously reported in poplar (see supporting information).

### Cell wall analyses

See supporting information.

## Author contributions

Da Lu performed plant harvesting, metabolite extractions, GC‐MS analyses, UPLC‐QTOF‐MS analyses, metabolomics, nontargeted metabolite identification and annotation, cell wall residue preparation, lignin monomer composition and content assessments, matrix polysaccharide composition and crystalline cellulose contents, and draft manuscript writing and research discussions. Xianghe Yuan performed nontargeted metabolite detection and provisional annotation, and draft manuscript section writing. Joaquim V. Marques performed metabolite provisional annotation and plant metabolomics, and draft manuscript writing. Sung‐Jin Kim performed hybrid poplar transformation and molecular biology analyses, and draft manuscript section writing. Randi Luchterhand and Barri Herman performed design, implementation, oversight and monitoring of all transgenic hybrid poplar field trials and plant harvesting, as well as in related draft manuscript section preparation. P. Pawan Chakravarthy performed synthesis of allyl/propenylphenol glucosides, and draft manuscript section writing. Laurence B. Davin and Norman G. Lewis responsibilities included research conception and discussions/guidance, completion of draft paper sections integration, multiple revisions and finalizing manuscript.

## Supporting information


**Figure S1** pK2GW7 Vector construction strategy.
**Figure S2** Transformation of hybrid poplar.
**Figure S3** Selected relative expression levels of *LtCAAT1* and *LtAPS1* and number of trees that flowered in each line.
**Figure S4** Selected relative expression levels of *LtCAAT1* and *LtPPS1* and number of trees that flowered in each line.
**Figure S5** Stem heights and diameters of transgenic hybrid poplar lines.
**Figure S6** Putative metabolites detected by non‐targeted UPLC metabolomic analyses of WT and transgenic LtAPS and LtPPS lines.
**Figure S7** Matrix polysaccharide content and composition.
**Figure S8** Hemicellulose and crystalline cellulose contents.
**Figure S9** Acetyl bromide lignin estimations.
**Figure S10** Estimated lignin compositions by thioacidolysis.
**Table S1** Composition of media and solutions used for transformation.
**Table S2** Gene specific primer sequences used for quantitative real‐time PCR.
**Tables S3 and S4** Targeted and non‐targeted UPLC metabolomics analyses of MeOH:H_2_O extracts of WT and LtAPS/LtPPS leaves in the negative ion mode.
**Appendix S1** Experimental procedures.
**Appendix S2** Non‐targeted metabolomics analyses and cell wall analysis results.
