# Supplementary material for: Eugenol specialty chemical production in transgenic poplar (Populus tremula × P. alba) field trials
Source: Plant Biotechnol J. 2017 Mar 7;15(8):970–81. doi: 10.1111/pbi.12692 (PMC5506655; doi:10.1111/pbi.12692)
Supplement: Supplementary file 1 — Figure S1 pK2GW7 Vector construction strategy. Figure S2 Transformation of hybrid poplar. Figure S3 Selected relative expression levels of LtCAAT1 and LtAPS1 and number of trees that flowered in each line. Figure S4 Selected relative expression levels of LtCAAT1 and LtPPS1 and number of trees that flowered in each line. Figure S5 Stem heights and diameters of transgenic hybrid poplar lines. Figure S6 Putative metabolites detected by non‐targeted UPLC metabolomic analyses of WT and transgenic LtAPS and LtPPS lines. Figure S7 Matrix polysaccharide content and composition. Figure S8 Hemicellulose and crystalline cellulose contents. Figure S9 Acetyl bromide lignin estimations. Figure S10 Estimated lignin compositions by thioacidolysis. Table S1 Composition of media and solutions used for transformation. Table S2 Gene specific primer sequences used for quantitative real‐time PCR. Tables S3 and S4 Targeted and non‐targeted UPLC metabolomics analyses of MeOH:H2O extracts of WT and LtAPS/LtPPS leaves in the negative ion mode. Appendix S1 Experimental procedures. Appendix S2 Non‐targeted metabolomics analyses and cell wall analysis results. [file PBI-15-970-s001.docx]

**Supplementary Information**

**Experimental Procedures**

**Chemicals**

For PCR and sequencing, custom oligonucleotide primers were synthesized by Invitrogen (San Jose, CA). PCR amplifications were performed on a C1000 Touch™ Thermal Cycler (Bio-Rad, Hercules, CA), whereas real time quantitative PCR analyses were carried out on a Mx3005P™ real time PCR system (Agilent Technologies, Santa Clara, CA). To isolate and purify plasmid DNA, Wizard Plus SV Miniprep Kit (Promega, Madison, WI) was used. For traditional molecular cloning, restriction enzymes were from New England Biolabs (Ipswich, MA), with a Rapid DNA Ligation Kit purchased from Roche. Quantitation of DNA and RNA analyses were done using a NanoDrop 2000 UV-Vis Spectrophotometer (Thermo Scientific).

For poplar transformation media, MS salts with vitamins, thidiazuron (TDZ), 6-benzylaminopurine (BAP), myo-inositol, 1-naphthalene acetic acid (NAA), 6-(γ,γ-dimethylallylamino)purine (2iP) and thiamine-HCl were purchased from Caisson Laboratory (North Logan, UT), whereas galactose, acetosyringone, ascorbic acid, nicotinic acid, pyridoxine HCl, L-cysteine, and D-pantothenic acid were from Sigma-Aldrich (St Louis, MO). Rifampicin, streptomycin and chloramphenicol were from Sigma (St Louis, MO), whereas spectinomycin was obtained from Research Products International Corp. (Mount Prospect, IL). MES (2-(*N*-morpholino) ethanesulfonic acid) buffer was from Research Organics (Cleveland, OH), with L-glutamine, biotin and kanamycin being purchased from ThermoFisher Scientific (San Jose, CA). Agar TC was from PhytoTechnology Laboratories^®^ (Shawnee Mission, KS), timentin from Agri-Bio (North Miami, FL), and sucrose was from J.T. Baker (Central Valley, PA).

Eugenol (**4**), acetobromo-α-D-glucose and Amberlite^®^ IR120 hydrogen form resin were purchased from Sigma-Aldrich (St Louis, MO). Ethyl acetate, hexanes, acetone, sodium hydroxide, sodium sulfate (anhydrous, granular, 12-60 mesh), dichloromethane, potassium carbonate (anhydrous, granular) and methanol were from J.T. Baker (Central Valley, PA), whereas silica gel was from Selecto Scientific (Suwanee, Georgia, USA).

Optima^TM^ grade H_2_O, acetonitrile and formic acid were purchased from ThermoFisher Scientific (San Jose, CA), whereas eugenol (**4**), isoeugenol (**8**), naringenin, *tert*-butyl methyl ether and benzyl methyl ether were acquired from Sigma-Aldrich (St. Louis, MO, USA), and leucine enkephalin was obtained from Waters (Milford, MA, USA).

**Chemical syntheses**

***Eugenol glucoside (6)***. Eugenol (**4)**, (1.0 g, 6.1 mmol) was added to a solution of NaOH (0.365 mg, 9.1 mmol) in acetone–H_2_O (45 mL, 1:1, v/v) at 0 °C. The resulting solution was then stirred for 1 h, followed by addition of acetobromoglucose (3.0 g, 7.3 mmol) in one portion. The reaction mixture was next stirred overnight at 0 °C, after which TLC (ethyl acetate: hexanes, 4:6, v/v) analysis indicated reaction completion. The acetone was then evaporated *in vacuo*, with the resulting aqueous layer diluted to 30 mL and extracted with ethyl acetate (25 mL × 3). The combined ethyl acetate layers were dried (anhydrous Na_2_SO_4_), then evaporated *in vacuo* to obtain the crude product (3.8 g), which was used directly without further purification.

Crude product (3.8 g) obtained above was next added to a solution of potassium carbonate (2.5 g, 18.1 mmol) in MeOH (50 mL) at 0 °C and stirred for 1 h, following which TLC (MeOH–CH_2_Cl_2_, 1:9, v/v) analysis indicated reaction completion. The resulting reaction mixture was then filtered, with the solid so obtained washed with MeOH (25 mL × 2). To the filtrate was next added pre-washed (MeOH) Amberlite-IR-120H^+^ until the pH was *ca.* 6.0-6.5. The resin was then removed by filtration, and washed with MeOH (25 mL × 2). The resulting filtrate was evaporated to dryness *in vacuo*, with the crude mixture purified by silica gel column chromatography, eluted with MeOH–CH_2_Cl_2_ (5:95, v/v), to afford eugenol glucoside (**6**, 572 mg, 24.8% yield). ^1^H NMR (Varian Mercury 300, CDCl_3_-CD_3_OD, 9:1) δ (ppm) 6.92 (d, J = 9.0 Hz, 1H), 6.64 (m, 2H), 5.97-5.71 (m, 1H), 5.0-4.9 (m, 2H), 4.71 (d, J = 7.2 Hz, 1H), 3.8-3.5 (m, 6H) 3.5-3.2 (m, 9H) (Ji and Yang, 2011; Mastelic *et al.*, 2004).

***Chavicol glucoside (5), p-anol glucoside (9) and isoeugenol glucoside (10).*** These were individually synthesized as described above for eugenol glucoside (**6**) from chavicol (**3**), *p*-anol (**7**) and isoeugenol (**8**), in 29, 18 and 37% yield (0.134, 0.83 and 0.182 g), respectively.

Chavicol glucoside (**5**). ^1^H NMR (CDCl_3_-CD_3_OD, 9:1) δ (ppm): 6.99 (d, J = 8.5 Hz, 2H), 6.9 (d, J = 8.5 Hz, 2H), 5.82 (m, 1H), 5.00 (m, 2H), 4.85-4.70 (m, 1H), 3.77-3.57 (m, 3H), 3.49-3.30 (m, 7H), 3.18-3.14 (d, J = 6.0Hz, 2H) (Ly *et al.*, 2002).

*p*-Anol glucoside (**9**). ^1^H NMR (CDCl_3_-CD_3_OD, 9:1) δ (ppm): 7.12 (d, J = 9.0 Hz, 2H), 6.89 (d, J = 9.0 Hz, 2H), 6.23 (d, J = 15.7 Hz, 1H), 6.12-5.90 (m, 1H), 4.8 1 (d, J = 7.1 Hz, 1H), 3.60-3.50 (m, 4H), 3.50-3.10 (m, 6H), 1.75 (d, J = 6.5 Hz, 3H) (Nakano *et al.*, 1988).

Isoeugenol glucoside (**10**). ^1^H NMR (CDCl_3_-CD_3_OD, 9:1) δ (ppm): 6.94 (d, J = 9.0 Hz, 1H), 6.81-6.66 (m, 2H), 6.19 (d, J=17.2 MHz, 1H), 6.09-5.91 (m, 1H), 4.69 (d, J = 7.3 Hz, 1H), 3.85-3.60 (m, 6H), 3.5-3.2 (m, 7H), 1.74 (d, J = 6.6 Hz, 3H) (Ji and Yang, 2011).

**Isolation and cloning of *LtCAAT1*, *LtAPS1* and *LtPPS1* coding genes and vector construction for heterologous** **expression**

*LtCAAT1*, *LtAPS1* and *LtPPS1* full-length cDNAs were isolated as described in Kim *et al.* (2014). Total RNA was obtained from *Larrea tridentata* mature leaf tissue using the Spectrum™ Plant Total RNA Kit (Sigma-Aldrich), with this then employed for first-strand cDNA synthesis using the SuperScript™ III First-Strand Synthesis System for RT-PCR (Invitrogen). With first-strand cDNA template, PCR amplification used the touch-down PCR protocol: 95 °C for 3 min, 10 cycles of denaturation at 95 °C (1 min), annealing (1 min) with temperature decreasing from 65 to 55 °C with each cycle, extension at 72 °C (2 min), 35 cycles of denaturation at 95 °C (1 min), annealing at 55 °C (1 min), extension at 72 °C (2 min), and final extension at 72 °C for 7 min by *PfuUltra* II Fusion HS DNA Polymerase (Agilent Technologies, Inc). *LtCAAT1*, *LtAPS1* and *LtPPS1* PCR products were individually cloned into the pENTR™/D-TOPO^®^ vector (Invitrogen), and their sequences confirmed. Each pENTR™/D vector construct was cloned into a pK2GW7 Gateway vector (Invitrogen), which included both *CaMV 35S* promoter and terminator regions by LR recombination of Gateway^®^ system, following the manufacturer’s instructions. For *LtCAAT1::LtAPS1* or *LtCAAT1::LtPPS1* double constructs, *p35S::LtAPS1::T35S* and *p35S::LtPPS1::T35S* gene cassettes were individually excised from the pK2GW7 vector using a traditional molecular cloning protocol, then fused together with the pK2GW7 vector construct harboring *p35S::LtCAAT1::T35S* (Figure S1) After verification by sequencing, the two different pK2GW7 vector constructs harboring either *p35S::LtCAAT1::T35S:: p35S::LtAPS1::T35S* or *p35S::LtCAAT1::T35S::p35S::LtPPS1::T35S* were individually utilized for transformation of *Agrobacterium* EHA105 by the freeze-thaw method (An *et al.*, 1988; Höfgen and Willmitzer, 1988).

**Hybrid poplar transformation**

Hybrid poplar (*Populus tremula x P. alba*) transformations were as for Filichkin *et al.* (2006). *Agrobacterium* EHA105 cells, transformed by pK2GW7 gateway vector constructs harboring either *p35S::LtCAAT1::T35S::p35S::LtAPS1::T35S* or *p35S::LtCAAT1:: T35S::p35S::LtPPS1::T35S*, were individually grown in 50 ml YEP medium containing 50 μg mL^–1^ rifampicin (Sigma), 50 μg mL^–1^ streptomycin (Sigma), 34 μg mL^–1^ chloramphenicol (Sigma), and 100 μg mL^–1^ spectinomycin (Research Products International Corp.) for 2 days at 28 °C. Cells were harvested by centrifugation at 2,000 × g for 20 min, and re-suspended in 30 mL *Agrobacterium* Induction Medium (IM, see Table S1). After re-pelleting by centrifugation, cells were re-suspended to a density of OD_600_ = 0.1 in *Agrobacterium* IM. Leaf discs, petioles and inter-nodal stem segments of 50 day old, *in vitro* grown, hybrid poplar INRA 717-1B4 (female, *P. tremula* × *P. alba*) were then incubated with the requisite *Agrobacterium* suspension for 1 h with gentle swirling. Corresponding explants were placed on Callus Induction Medium 1 (CIM1, Table S1) and co-cultivated at room temperature in the dark for 3 days. Inoculated explants were washed twice with 50 mL sterile H_2_O four times and 50 mL Wash Solution (Table S1), with explants replanted and co-cultivated on Callus Induction Medium 2 (CIM2, Table S1) containing antibiotics (kanamycin and timentin) in the dark for 21 days (Figure S2a).

Shoot induction from each transformed callus was carried out on Shoot Induction Medium (SIM, Table S1) at room temperature under light for 2–3 months (Figure S2b and S2c). Induced shoots were elongated by culturing explants on Shoot Elongation Medium (SEM, Table S1) for 1–2 months (Figure S2d and S2e). For root induction, well-developed regenerated shoots for each transformant were individually transferred on Root Induction Medium (RIM, Table S1) and grown for 2–3 months until stems reached 8–10 cm in length (Figure S2f and S2g).

Regenerated transgenic plants (8–10 cm stem) were next individually potted in soil, covered with a plastic dome to maintain high humidity, and grown under light for 2–3 weeks (Figure S2h), and finally moved into a growth chamber (without plastic dome) and grown for an additional 3–4 weeks (Figure S2i), after which 212 *LtCAAT1::LtAPS1* and 178 *LtCAAT1::LtPPS1* transgenic poplars were transferred to the greenhouse (Figure S2j).

**Total RNA isolation and quantitative real-time PCR for selection of transgenic poplar**

Leaves (taken from 12^th^ node from the apical meristem) were individually harvested from 3-4 month greenhouse grown trees for each transgenic (*LtCAAT1::LtAPS1* or *LtCAAT1::LtPPS1*) line and WT, and immediately frozen in liquid nitrogen. Two to three leaf disks (~ 1 cm diameter) were ground using a TissueLyser II with Stainless Steel Beads, 5 mm (Qiagen) at 30 Hz frequency for 1 min. Total RNA was individually isolated using a Spectrum™ Plant Total RNA Kit (Sigma-Aldrich) following the manufacturer's instructions. With 1 μg of each DNase I-treated total RNA and oligo(dT)_20_ primer, first strand cDNA was individually synthesized using the SuperScript^®^ III First-Strand Synthesis System for RT-PCR (Invitrogen) according to the manufacturer's instructions. After RNase H treatment, each cDNA synthesis reaction (1:5 diluted with nuclease-free H_2_O) was utilized for qRT-PCR analyses with a final concentration of 0.5 μM of each gene specific primer set (Table S2) and Platinum^®^ SYBR^®^ Green qPCR SuperMix-UDG (Invitrogen).

qRT-PCR was performed using a Mx3005P^™^ qPCR System (Agilent Technologies, Inc.) as follows: 95 °C for 10 min, 40 cycles of denaturation at 95 °C for 30 sec, annealing at 55 °C for 1 min and extension at 72 °C for 30 sec. Each PCR reaction performed in triplicate was normalized to the *PtACT2* gene (Table S2) (Brunner *et al.*, 2004). Both WT poplar and *L. tridentata* were used as controls. For *L. tridentata*, its *ACTIN2* gene was used for qRT-PCR normalization. *LtCAAT1*, *LtAPS1* and *LtPPS1* gene expression levels in *L. tridentata* were set to 1.

**Extraction protocol standardization/calibration**

To ensure accuracy of determinations of metabolites through harvest, storage and extraction processes, recovery experiments were performed by adding authentic standards before extraction. For each of the 11 lines, six replicates were analyzed.

For GCMS analyses, calibration curves for eugenol (**4**), chavicol (**3**) and isoeugenol (**8**) were generated for quantification in the same biological matrix. The extraction solvent utilized *tert*-butyl methyl ether containing 0.5 mM benzyl methyl ether IS to define the instrument response to concentration relationship. Peak areas of targeted sample components were normalized to the IS and quantified using calibration curves also normalized to the IS. (IS recovery was usually in the order of > 85%.)

Calculations used for quantification are indicated:

$$metabolite fresh weight \%=\frac{Peak area ofmetabolite}{Coefficient of calibration curve}\times Extraction ratio\times MW\times\frac{IS peak area of blank}{IS peak area of each sample} \times100\%$$

With:

Extraction ratio: 2 µL mg tissue^–1^

MW: molecular weight of metabolite, g mol^–1^

For UPLC-QTOF-MS analyses, calibration curves for the allyl/propenylphenol glucosides (**5**, **6**, **9** and **10**) were generated with normalization to the internal standard (IS, naringenin).

Calculations used for quantification are indicated:

$$metabolite dry weight \%=\frac{Peak area ofmetabolite}{Coefficient of calibration curve}\times Extraction ratio\times\frac{IS peak area of blank}{IS peak area of each sample} \times100\%$$

With:

Extraction ratio: 20 µL mg tissue^–1^

**Cell wall residue preparation**

Branches were individually freeze-dried, cut into small pieces, and ground to a fine powder with a ball mill (Retsch PM400). The milled samples were then treated with different solvents in five successive extractions: toluene-EtOH (50:50, v/v, × 2), EtOH, and distilled H_2_O (× 2) at room temperature. Each sample was mixed at a ratio of 50 mL solvent to *ca.* 5 g dry tissue powder, then stirred for eight hours and centrifuged (20,000 *g*, 15 min, 4 °C) for each extraction. After each final extraction step, cell wall residues were individually freeze-dried and stored at room temperature for subsequent lignin analyses.

**Thioacidolysis analyses for lignin monomer composition and content assessments**

Thioacidolysis determinations (Blee *et al.*, 2001; Rolando *et al.*, 1992) were carried out in triplicate as described in Foster *et al.* (2010a) with the following modifications. To ~2 mg of each cell wall residue in a screw-capped glass tube, dioxane (350 µL), EtSH (40 µL) and BF_3_ (10 µL) were added. After 4 h at 100 °C, each reaction was stopped by cooling for 5 min on ice, with 0.4 M NaHCO_3_ (300 µL) and CH_2_Cl_2_ (200 µL) containing tetracosane (5 mg mL^–1^) added to each sample. Each suspension was next vortexed for a few seconds followed by addition of H_2_O (300 µL) and EtOAc (500 µL). After vortexing again for a few seconds, a portion of each EtOAc layer (300 µL) was transferred into a 2 mL screw-capped glass tube. For TMS derivatization, CH_2_Cl_2_ (200 µL), pyridine (20 µL) and N,O-bis(trimethylsilyl) acetamide (BSTFA, 100 µL) were added to each tube, with the whole individually incubated for 2 hours at 25 °C. Each sample was then subjected to GC-MS analyses using a RESTEK Rxi^®^-5MS column (Foster *et al.*, 2010a).

**Acetyl bromide lignin content estimations**

Acetyl bromide lignin content estimations (Blee *et al.*, 2001; Iiyama and Wallis, 1988) were carried out in triplicate as described in Foster *et al.* (2010a), with the absorption of each solution determined at 280 nm using a UV/VIS spectrometer.

**Matrix polysaccharide compositions and crystalline cellulose contents**

Matrix polysaccharide composition (Albersheim *et al.*, 1967) analyses were carried out in triplicate as described in Foster *et al.* (2010b) with GC-MS analyses, with crystalline cellulose contents (Updegraff, 1969) estimated with the absorption of each sample tested at 625 nm using UV/VIS spectrometer.

**Non-targeted Metabolomics Analyses Results**

A characteristic class of compounds in poplar are compounds derived from salicylate. One of these, possibly tremulacinol (Ishikawa *et al.*, 2004), displayed relatively small increases in amounts in the engineered plants, i.e. *ca*. 1.31 and 1.44 fold increases (p = 0.048 and 0.057) (Supplemental Tables S3/S4 and Figure S6d). Two putative hydroxycinnamyl-quinates, with the same mass spectra but different retention times, were also detected and putatively annotated as *p*-coumaroyl-quinic acid-1 and *p*-coumaroyl-quinic acid-2. Both compounds displayed different trends: one was slightly decreased in amounts in the transgenics (*ca.* 0.82 and 0.58 fold changes, p = 0.199 and 0.018), whereas the other had a slight increase (*ca.* 1.05 and 1.47 fold changes, p = 0.291 and 0.019) in the LtAPS and LtPPS lines, respectively (Supplemental Tables S3/S4 and Figure S6b). We also putatively annotated a blumenol B glucoside which was modestly increased in amount in the transgenics, with 1.26 and 1.45 fold changes (p = 0.067 and 0.015), respectively (Supplemental Tables S3/S4 and Figure S6c).

There were also eight flavonoids detected and provisionally quercetin-*O*-hexoside-*O*-pentosylhexoside-*O*-hexoside like, taxifolin like, quercetin-*O*-hexoside-*O*-rhamnoside-*O*-hexoside like, isorhamnetin-*O*-rutinoside-*O*-pentose like, kaempferol-dihexosylpentoside like, quercetin-glucuronide like, isorhamnetin-pentosylglucuronide like and kaempferol-glucoside like in leaf tissues (Supplemental Tables S3/S4 and Figure S6a). Two of these, the kaempferol-dihexosylpentoside like and isorhamnetin-pentosylglucuronide like derivatives, were significantly reduced in amounts in the transgenic lines relative to WT (*ca.* 0.29/0.31, p = 0.009/0.0095 and 0.42/0.44, p = 0.007/0.002), respectively. Amounts of the putative quercetin-*O*-hexoside-*O*-pentosylhexoside-*O*-hexoside like derivative were also decreased in fold change amounts (to 0.39/0.65), with the most pronounced being in the LtPPS line. By contrast, the taxifolin like and quercetin-*O*-hexoside-*O*-rhamnoside-*O*-hexoside like derivatives showed overall fold change increases of 1.20/1.64 and 1.67/2.01, whereas the quercetin glucuronide and isorhamnetin-*O*-rutinoside-*O*-pentose like moieties were lesser affected (0.98/1.27 and 0.74/0.80 fold changes).

**Cell Wall Analysis Results**

It was of interest to establish if there were any significant effects in cell wall composition in branches of the various WT and LtAPS and LtPPS transformed poplar lines. This was largely to determine whether amounts and compositions of cell wall carbohydrates (cellulose, hemicelluloses) and lignins differed in the various lines.

**Matrix Polysaccharides**

Matrix polysaccharide compositions were analyzed, in terms of sugar (monosaccharide) composition, as well as hemicellulose and cellulose contents (Figures S6 and S7). Cell wall residues (*ca.* 2 mg) were used for sugar analyses, with inositol employed as IS. After alditol acetate derivatization, alditol acetylation and alditol acetate extraction, samples were analyzed using GCMS. Monosaccharides were identified/quantified by mass profiles and/or retention times of standards, with cellulose contents obtained using the remaining pellets (Figures S6a and S6b). With Saeman hydrolysis and a colorimetric anthrone assay, amounts were calculated according to absorbances at 625 nm. To obtain the standard curve, 2 mg·mL^–1^ glucose stock (dissolved in water, stored at 0 °C) was used. After calibration, average amounts of total sugars were quantified for WT, LtAPS and LtPPS lines as 221.22 ± 11.06 µg·mg^–1^, 192.98 ± 9.65 µg·mg^–1^ and 223.29 ± 11.16 µg·mg^–1^, respectively (Figure S6a). Results obtained were similar for WT and LtPPS lines, while LtAPS lines had a reduced total sugar content by *ca*. 13 ± 5 %. Average hemicellulose contents showed similar values for WT and LtPPS as 23.48 ± 5 and 23.00 ± 5 %, respectively, whereas LtAPS lines had lower average contents (20.42 ± 5 %) by *ca.* 13 ± 5 % (Figure S7a). Estimated cellulose contents for WT, LtAPS and LtPPS were 42.61 ± 5 %, 47.79 ± 5 % and 43.12 ± 5 %, respectively, while the LtAPS lines apparently had slightly increased cellulose contents by *ca*. 12 ± 5 % (Figure S7b). The significance, if any, of these differences is not known at present.

**Lignin Analyses**

To examine whether so-called “abnormal lignins” were produced using, at least in part, allyl/propenyl phenol precursors that would be detected in thioacidolysis determinations (which cleave β-*O*-aryl functionalities in lignins). Various tissues were subjected to lignin composition and content analyses, using thioacidolysis, as well as AcBr gross lignin estimates (Foster *et al.*, 2010a), methods, respectively. For the AcBr lignin estimations of gross lignin contents (Figure S8), the WT lines had on average AcBr lignin contents of 26 ± 5% on average, whereas the LtAPS and LtPPS were 26 ± 5 and 24 ± 5 % respectively. These differences were not considered to be significant.

Thioacidolysis allowed for determination of the nature/content of the monomeric moieties released, i.e. in terms of *p*-hydroxyphenol- (H), guaiacyl- (G) and syringyl- (S) units. For the WT lines, the averaged results of H, G and S units were 4.35 ± 0.22 µmol·
g^–1^, 565.90 ± 28.29 µmol·g^–1^ and 498.88 ± 24.94 µmol·g^–1^ (total ~1069.13 µmol·g^–1^), whereas in the LtAPS lines, averaged results gave 4.58 ± 0.23 µmol·g^–1^, 546.60 ± 27.33 µmol·g^–1^ and 322.16 ± 16.11 µmol·g^–1^ (total ~873.34 µmol·g^–1^), respectively (Figure S9). That is, amounts of released S units were apparently decreased by *ca*. 35 ± 5 %, while amounts of H and G units were essentially unchanged.

Interestingly, in LtPPS lines, the averaged results of H, G and S units were 2.17 ± 0.11 µmol·g^–1^, 429.68 ± 21.48 µmol·g^–1^ and 288.97 ± 14.45 µmol·g^–1^ (total ~720.82 µmol·g^–1^), these being reduced by *ca.* 50 ± 5 %, 24 ± 5 % and 42 ± 5 %, respectively, relative to WT.

Importantly, no other new peaks were observed in the thioacidolysis derived GC chromatograms, indicating no detection of any “abnormal lignin” being present. While the reasons for the observed differences in overall monomer release levels are unknown, they may simply reflect growth/development/sampling differences.

**References**

Albersheim, P., Nevins, D.J., English, P.D. and Karr, A. (1967) A method for the analysis of sugars in plant cell-wall polysaccharides by gas-liquid chromatography. *Carbohydr. Res.* **5**, 340-345.

An, G., Ebert, P.R., Mitra, A. and Ha, S.B. (1988) Binary vectors. In: *Plant Molecular Biology Manual* (Gelvin, S.B., Schilperoort, R.A. and Verma, D.P.S. eds), Vol. A3, pp. 29-47. Kluwer Academic Publishers: Dordrecht.

Blee, K., Choi, J.W., O’Connell, A.P., Jupe, S.C., Schuch, W., Lewis, N.G. and Bolwell, G.P. (2001) Antisense and sense expression of cDNA coding for CYP73A15, a class II cinnamate-4-hydroxylase, leads to a delayed and reduced production of lignin in tobacco. *Phytochemistry* **57**, 1159-1166.

Brunner, A.M., Yakovlev, I.A. and Strauss, S.H. (2004) Validating internal controls for quantitative plant gene expression studies. *BMC Plant Biol.* **4**, 14.

Filichkin, S.A., Meilan, R., Busov, V.B., Ma, C., Brunner, A.M. and Strauss, S.H. (2006) Alcohol-inducible gene expression in transgenic *Populus*. *Plant Cell Rep.* **25**, 660-667.

Foster, C.E., Martin, T.M. and Pauly, M. (2010a) Comprehensive compositional analysis of plant cell walls (lignocellulosic biomass). Part I: Lignin. *J. Vis. Exp.* **37**, e1745.

Foster, C.E., Martin, T.M. and Pauly, M. (2010b) Comprehensive compositional analysis of plant cell walls (lignocellulosic biomass). Part II: Carbohydrates. *J. Vis. Exp.* **37**, e1837.

Höfgen, R. and Willmitzer, L. (1988) Storage of competent cells for *Agrobacterium* transformation. *Nucleic Acids Res.* **16**, 9877.

Iiyama, K. and Wallis, A.F.A. (1988) An improved acetyl bromide procedure for determining lignin in woods and wood pulps. *Wood Sci. Technol.* **22**, 271-280.

Ishikawa, T., Nishigaya, K., Takami, K., Uchikoshi, H., Chen, I.-S. and Tsai, I.-L. (2004) Isolation of salicin derivatives from *Homalium cochinchinensis* and their antiviral activities. *J. Nat. Prod.* **67**, 659-663.

Ji, J.-l. and Yang, S.-g. (2011) Synthesis of eugenol and isoeugenol-β-D-glucoside. *Journal of Shanghai Institute of Technology* **11**, 46-50.

Kim, S.-J., Vassão, D.G., Moinuddin, S.G.A., Bedgar, D.L., Davin, L.B. and Lewis, N.G. (2014) Allyl/propenyl phenol synthases from the creosote bush and engineering production of specialty/commodity chemicals, eugenol/isoeugenol, in *Escherichia coli*. *Arch. Biochem. Biophys.* **541**, 37-46.

Ly, T.N., Yamauchi, R., Shimoyamada, M. and Kato, K. (2002) Isolation and structural elucidation of some glycosides from the rhizomes of smaller galanga (*Alpinia officinarum* Hance). *J. Agric. Food Chem.* **50**, 4919-4924.

Mastelic, J., Jerkovic, I., Vinkovic, M., Dzolic, Z. and Vikic-Topic, D. (2004) Synthesis of selected naturally occurring glucosides of volatile compounds. Their chromatographic and spectroscopic properties. *Croat. Chem. Acta* **77**, 491-500.

Nakano, K., Nishizawa, K., Takemoto, I., Murakami, K., Takaishi, Y. and Tomimatsu, T. (1988) Flavonol and phenylpropanoid glycosides from *Lilium cordatum*. *Phytochemistry* **28**, 301-303.

Rolando, C., Monties, B. and Lapierre, C. (1992) Thioacidolysis. In: *Methods in Lignin Chemistry* (Lin, S.Y. and Dence, C.W. eds), pp. 334–349. Springer-Verlag: Berlin.

Updegraff, D.M. (1969) Semi-micro determination of cellulose in biological materials. *Anal. Biochem.* **32**, 420-424.

**Table S1**. Composition of media and solutions used for *Agrobacterium*-mediated transformation of *Populus tremula* × *P. alba* INRA 717-1B4

| Medium / Solution | Composition |
| --- | --- |
| IM | MS salt with vitamins, 10 mM galactose, 1.28 mM MES, and  50 μM acetosyringone; pH 5.0 |
| Wash Solution | MS salt with vitamins, 1.0 μM 1-naphthalene acetic acid (NAA), 1.0 μM 6-benzylaminopurine (BAP), 1.0 μM 6-(γ,γ-dimethylallyl-amino)purine (2iP), 0.25 g L^–1^ ascorbic acid, and 0.2 g L^–1^ timentin; pH 5.8 |
| CIM1 | MS salt without vitamins, 30 g L^–1^ sucrose, 0.25 g L^–1^ MES,  0.1 g L^–1^ *myo*-inositol, 10 μM NAA, 0.2 g L^–1^ L-glutamine,  5 μM 2iP, FV* vitamins, and 5 g L^–1^ agar TC; pH 5.8 |
| CIM2 | CIM1 medium containing 50 mg L^–1^ kanamycin and  0.2 g L^–1^ timentin |
| SIM | MS salt without vitamins, 30 g L^–1^ sucrose, 0.25 g L^–1^ MES,  0.1 g L^–1^ *myo*-inositol, 0.2 μM thidiazuron (TDZ),  0.2 g L^–1^ L-glutamine, FV vitamins, 0.1 g L^–1^ kanamycin,  0.2 g L^–1^ timentin, and 5 g L^–1^ agar TC; pH 5.8 |
| SEM | MS salt without vitamins, 30 g L^–1^ sucrose, 0.25 g L^–1^ MES,  0.1 g L^–1^ *myo*-inositol, 0.1 μM 6-benzylaminopurine (BAP),  0.2 g L^–1^ L-glutamine, FV vitamins, 0.1 g L^–1^ kanamycin,  0.2 g L^–1^ timentin, and 5 g L^–1^ agar TC; pH 5.8 |
| RIM | ½ MS salt without vitamins, 20 g L^–1^ sucrose, 0.25 g L^–1^ MES,  0.1 g L^–1^ *myo*-inositol, 0.5 μM indole-3-butyric acid (IBA),  0.2 g L^–1^ L-glutamine, FV vitamins, 0.25 g L^–1^ kanamycin,  0.1 g L^–1^ timentin, and 5 g L^–1^ agar TC; pH 5.8 |
| *FV vitamins (100 ×) | 0.1 g L^–1^ nicotinic acid, 0.1 g L^–1^ pyridoxine HCl,  0.1 g L^–1^ L-cysteine, 0.1 g L^–1^ D-pantothenic acid,  0.1 g L^–1^ thiamine-HCl, and 4.1 μM biotin |

**Table S2**. Gene specific primer sequences used for quantitative real-time PCR

| Primer name | Primer sequence |
| --- | --- |
| PtriActin2-For | 5′-GCCGATGCCGAGGATATTCAAC-3′ |
| PtriActin2-Rev | 5′-ATCACCTGCAAACCCAGCCTTCAC-3′ |
| LtCAAT1-qP-For | 5′-GGTGGAGGCTCACCGACCAG-3′ |
| LtCAAT1-qP-Rev | 5′-CACCACAAAACCCGGTCCATC-3′ |
| LtAPS1-qP-For | 5′-GCTGCTATCAAAGCGGCTGGA-3′ |
| LtAPS1-qP-Rev | 5′-CAGCATGGGTTCGGTCAACG-3′ |
| LtPPS1-qP-For | 5′-GACTGGAAGGACCCTCCGAAGA-3′ |
| LtPPS1-qP-Rev | 5′-AGGGACGGGCAGGGTTTCAG-3′ |

**Table S3**. Targeted and non -targeted UPLC metabolomics analyses of MeOH:H_2_O extracts of WT and LtAPS leaves in the negative ion mode.

| **Feature** | ***m/z*** | **Retention time (min)** | **Calculated mass** | **Error*(ppm)** | **Ion** | **Annotation** | | **Category** | **p-Value**  **WT/**  **LtAPS** | **Fold change**  **LtAPS/**  **WT** |
| --- | --- | --- | --- | --- | --- | --- | --- | --- | --- | --- |
| **Targeted** | | | | | | | | | | |
| M371T1049 | 371.1343 | 17.48 | 371.1342 | 0.17 | [M+HCOO]^–^ | Eugenol glucoside (**6**) | | Phenolic glucoside | 9.54×10^-9^ | **∞** |
| M341T1034 | 341.1239 | 17.23 | 341.1236 | 0.77 | [M+HCOO]^–^ | Chavicol glucoside (**5**) | | Phenolic glucoside | 3.32×10^-5^ | **∞** |
| **Non-targeted** | | | | | | | | | | |
| M503T966 | 503.1767 | 16.10 | 503.1765 | 0.52 | [M+HCOO]^–^ | Eugenol-pentoside-hexoside like | | Phenolic glucoside | 1.72×10^-6^ | **∞** |
| M303T579 | 303.0503 | 9.65 | 303.0505 | 0.57 | [M–H]^–^ | Taxifolin like |  | Flavonoid | 0.2655 | 1.20 |
| M771T595 | 771.1982 | 9.92 | 771.1984 | 0.07 | [M–H]^–^ | Quercetin-*O*-hexoside-*O*-rhamnoside-*O*-hexoside like | | Flavonoid | 0.0044 | 1.67 |
| M477T863 | 477.0671 | 14.39 | 477.0669 | 0.44 | [M–H]^–^ | Quercetin-glucuronide like | | Flavonoid | 0.9398 | 0.98 |
| M447T934 | 447.0925 | 15.56 | 447.0927 | 0.53 | [M–H]^–^ | Kaempferol-glucoside like | | Flavonoid | 0.1281 | 1.45 |
| M433T774 | 433.2074 | 12.91 | 433.2074 | 0.03 | [M+HCOO]^–^ | Blumenol B glucoside like | | Blumenol A/B | 0.0673 | 1.26 |
| M529T1271 | 529.1705 | 21.18 | 529.1710 | 0.87 | [M–H]^–^ | Tremulacinol like | | Salicylate | 0.0476 | 1.31 |
| M337T702 | 337.0923 | 11.70 | 337.0923 | 0.19 | [M–H]^–^ | *p*-Coumaroyl quinic acid-2 like | | Hydroxycinnamyl-quinates | 0.2908 | 1.05 |
| M337T589 | 337.0924 | 9.82 | 337.0923 | 0.07 | [M–H]^–^ | *p*-Coumaroyl quinic acid-1 like | | Hydroxycinnamyl-quinates | 0.1988 | 0.82 |
| M903T538 | 903.2391 | 8.97 | 903.2406 | 1.68 | [M–H]^–^ | Quercetin-*O*-hexoside-*O*-pentosylhexoside-*O*-hexoside like | | Flavonoid | 0.0124 | 0.39 |
| M755T810 | 755.2043 | 13.50 | 755.2035 | 1.13 | [M–H]^–^ | Isorhamnetin-*O*-rutinoside-*O*-pentose like | | Flavonoid | 0.0016 | 0.74 |
| M741T839 | 741.1884 | 13.98 | 741.1878 | 0.81 | [M–H]^–^ | Kaempferol-dihexosylpentoside like | | Flavonoid | 0.0090 | 0.29 |
| M623T869 | 623.1258 | 14.48 | 623.1248 | 1.50 | [M–H]^–^ | Isorhamnetin-pentosylglucuronide like | | Flavonoid | 0.0007 | 0.42 |
| M359T477 | 359.0976 | 7.95 | 359.0978 | 0.52 | [M–H]^–^ | Syringic acid hexoside derivative | | Syringic acid | 0.1431 | 0.83 |

* Error (ppm) is calculated as follows: $\frac{measured mass-calculated mass}{calculated mass}$. Calculated mass and *m/z* in Table only show the data to the nearest 4 decimal points, whereas the error (ppm) is calculated based on the actual data obtained.

**Table S4**. Targeted and non -targeted UPLC metabolomics analyses of MeOH:H_2_O extracts of WT and LtPPS leaves in thenegative ion mode.

| **Feature** | ***m/z*** | **Retention time (min)** | **Calculated mass** | **Error*(ppm)** | **Ion** | **Annotation** | | **Category** | **p-Value**  **WT/**  **LtAPS** | **Fold change**  **LtAPS/**  **WT** |
| --- | --- | --- | --- | --- | --- | --- | --- | --- | --- | --- |
| **Targeted** | | | | | | | | | | |
| M371T1049 | 371.1343 | 17.48 | 371.1342 | 0.17 | [M+HCOO]^–^ | Eugenol glucoside (**6**) | | Phenolic glucoside | 7.55×10^-8^ | **∞** |
| M341T1034 | 341.1239 | 17.23 | 341.1236 | 0.77 | [M+HCOO]^–^ | Chavicol glucoside (**5**) | | Phenolic glucoside | 6.25×10^-6^ | **∞** |
| **Non-targeted** | | | | | | | | | | |
| M503T966 | 503.1767 | 16.10 | 503.1765 | 0.52 | [M+HCOO]^–^ | Eugenol-pentoside-hexoside like | | Phenolic glucoside | 7.35×10^-6^ | **∞** |
| M303T579 | 303.0503 | 9.65 | 303.0505 | 0.57 | [M–H]^–^ | Taxifolin like |  | Flavonoid | 0.0003 | 1.64 |
| M771T595 | 771.1982 | 9.92 | 771.1984 | 0.07 | [M–H]^–^ | Quercetin-*O*-hexoside-*O*-rhamnoside-*O*-hexoside like | | Flavonoid | 0.0044 | 0.0007 |
| M477T863 | 477.0671 | 14.39 | 477.0669 | 0.44 | [M–H]^–^ | Quercetin-glucuronide like | | Flavonoid | 0.9398 | 0.0003 |
| M447T934 | 447.0925 | 15.56 | 447.0927 | 0.53 | [M–H]^–^ | Kaempferol-glucoside like | | Flavonoid | 0.1281 | 0.0029 |
| M433T774 | 433.2074 | 12.91 | 433.2074 | 0.03 | [M+HCOO]^–^ | Blumenol B glucoside like | | Blumenol A/B | 0.0673 | 0.0145 |
| M529T1271 | 529.1705 | 21.18 | 529.1710 | 0.87 | [M–H]^–^ | Tremulacinol like | | Salicylate | 0.0476 | 0.0571 |
| M337T702 | 337.0923 | 11.70 | 337.0923 | 0.19 | [M–H]^–^ | *p*-Coumaroyl quinic acid-2 like | | Hydroxycinnamyl-quinates | 0.2908 | 0.0190 |
| M337T589 | 337.0924 | 9.82 | 337.0923 | 0.07 | [M–H]^–^ | *p*-Coumaroyl quinic acid-1 like | | Hydroxycinnamyl-quinates | 0.0179 | 0.58 |
| M903T538 | 903.2391 | 8.97 | 903.2406 | 1.68 | [M–H]^–^ | Quercetin-*O*-hexoside-*O*-pentosylhexoside-*O*-hexoside like | | Flavonoid | 0.1464 | 0.65 |
| M755T810 | 755.2043 | 13.50 | 755.2035 | 1.13 | [M–H]^–^ | Isorhamnetin-*O*-rutinoside-*O*-pentose like | | Flavonoid | 0.0352 | 0.80 |
| M741T839 | 741.1884 | 13.98 | 741.1878 | 0.81 | [M–H]^–^ | Kaempferol-dihexosylpentoside like | | Flavonoid | 0.0095 | 0.31 |
| M623T869 | 623.1258 | 14.48 | 623.1248 | 1.50 | [M–H]^–^ | Isorhamnetin-pentosylglucuronide like | | Flavonoid | 0.0022 | 0.44 |
| M359T477 | 359.0976 | 7.95 | 359.0978 | 0.52 | [M–H]^–^ | Syringic acid hexoside derivative | | Syringic acid | 0.0046 | 0.71 |

For explanations of *, see Table S3.


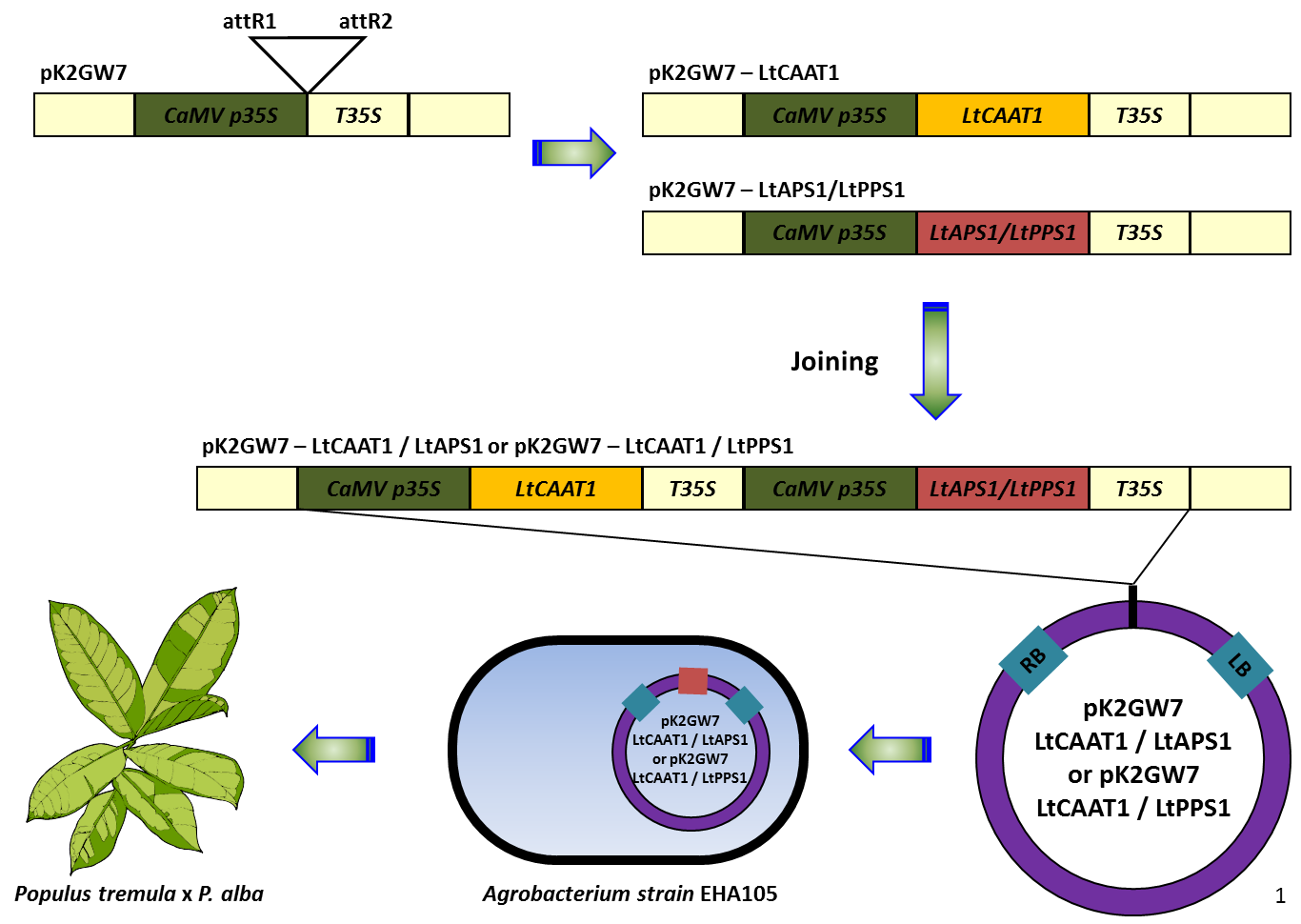


**Figure S1**. pK2GW7 Vector construction strategy for hybrid poplar (*P. tremula* × *P. alba*, INRA 717-1B4) transformation.


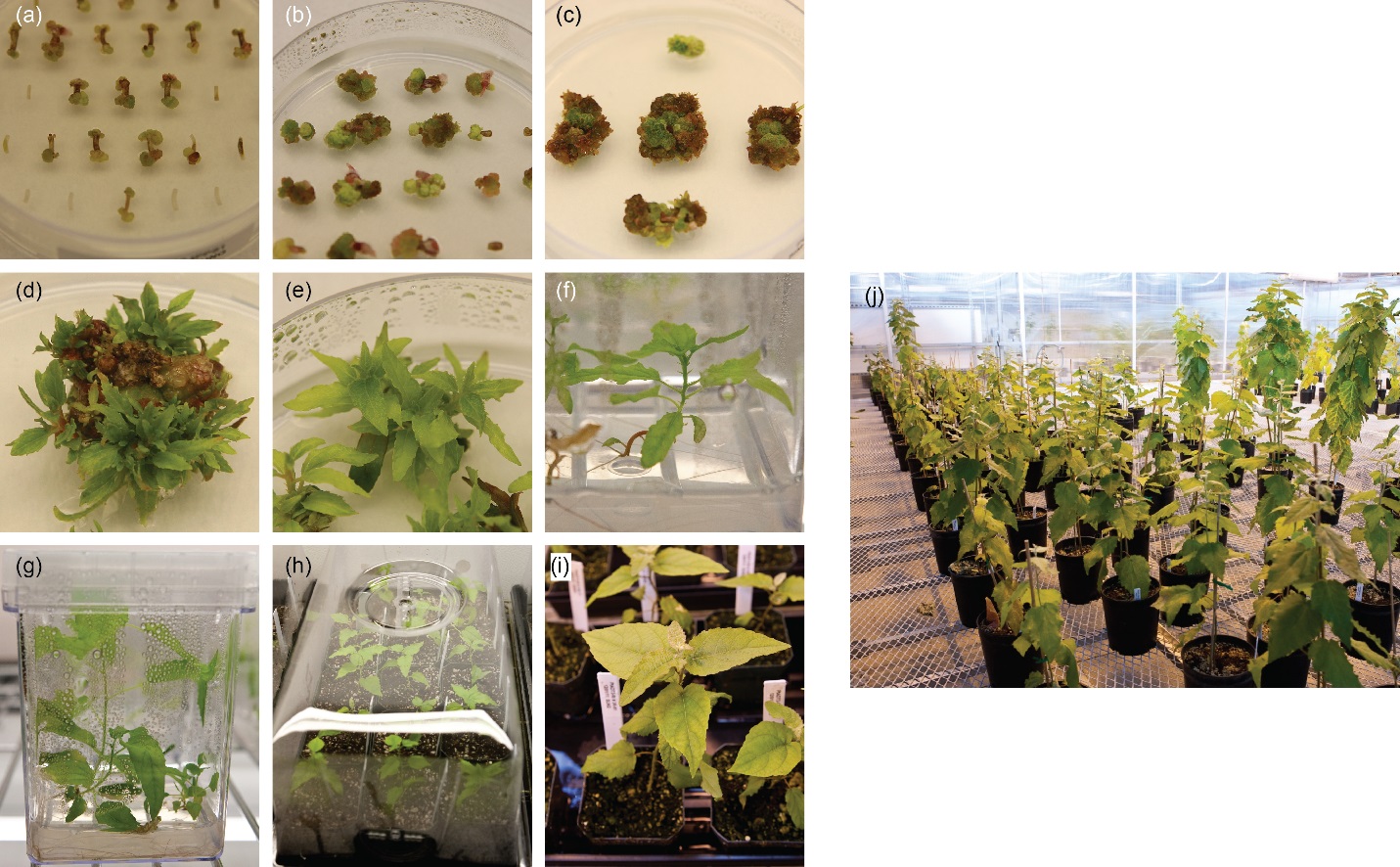


**Figure S2**. Transformation of hybrid poplar. (a – c) Explants on shoot induction medium 30 (a), 45 (b) and 60 (c) days from callus induction. (d, e) Explants on shoot elongation medium 90 (d), and 120 (e) days from callus induction. (f, g) Explants on root induction medium 140 (f), and 180 (g) days from callus induction. (h) Transgenic trees transferred to soil and maintained under a plastic dome to control humidity levels for ~2 – 3 weeks. (i) Transgenic trees transferred to an environmental growth chamber for 3 – 4 weeks. (j) Transgenic trees in greenhouse.


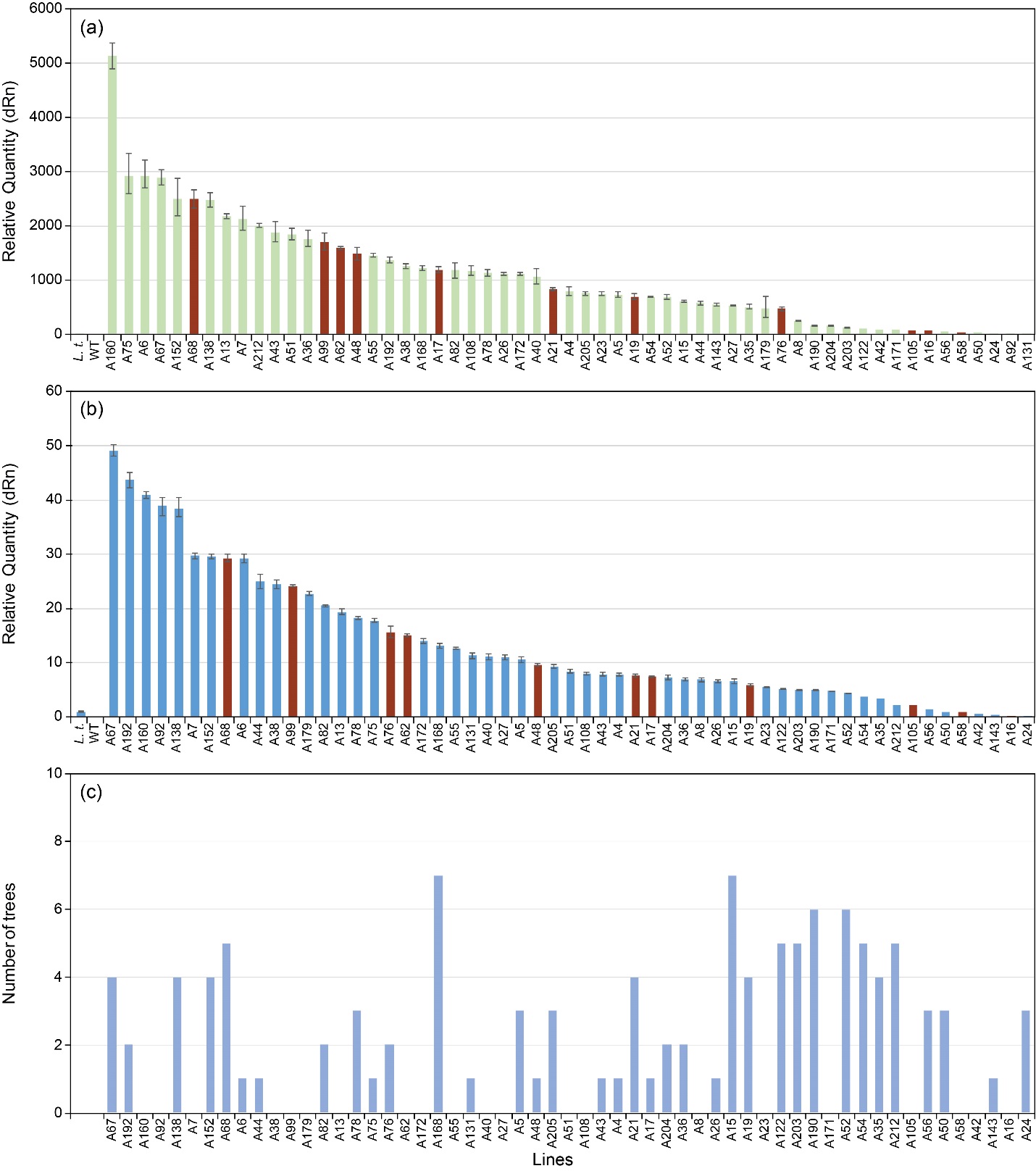


**Figure S3.** Selected relative expression levels of *LtCAAT1* (a) and *LtAPS1* (b) in hybrid poplar transformed with the *p35S::LtCAAT1::T35S::p35S::LtAPS1::T35S* construct as determined by real-time PCR. Values are expressed relative to their corresponding expression level in *Larrea tridentata* (*L. t.*) which was set at 1. No *LtAPS1* and *LtCAAT1* gene expression was detected in WT *P. tremula* × *P. alba*. Lines shown in red in (a) and (b) were harvested in August 2014.
(c) Number of trees that flowered in each line during Spring 2016.


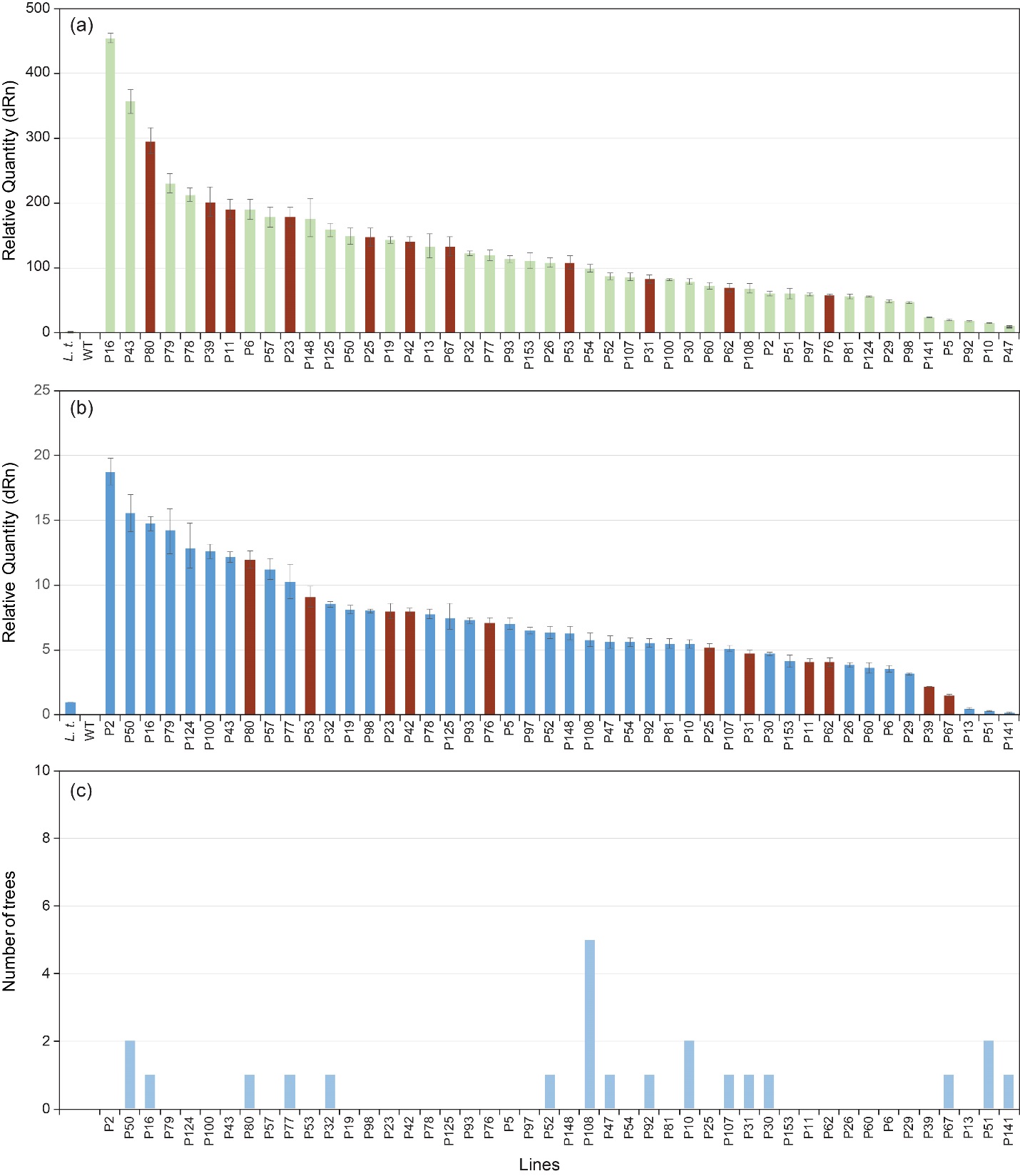


**Figure S4.** Selected relative expression levels of *LtCAAT1* (a) and *LtPPS1* (b) in hybrid poplar transformed with the *p35S::LtCAAT1::T35S::p35S::LtPPS1::T35S* construct as determined by real-time PCR. Values are expressed relative to their corresponding expression level in *Larrea tridentata* (*L. t.*) which was set at 1. No *LtPPS1* and *LtCAAT1* gene expression was detected in WT *P. tremula* × *P. alba*. Lines shown in red in (a) and (b) were harvested in August 2014.
(c) Number of trees that flowered in each line during Spring 2016.

**
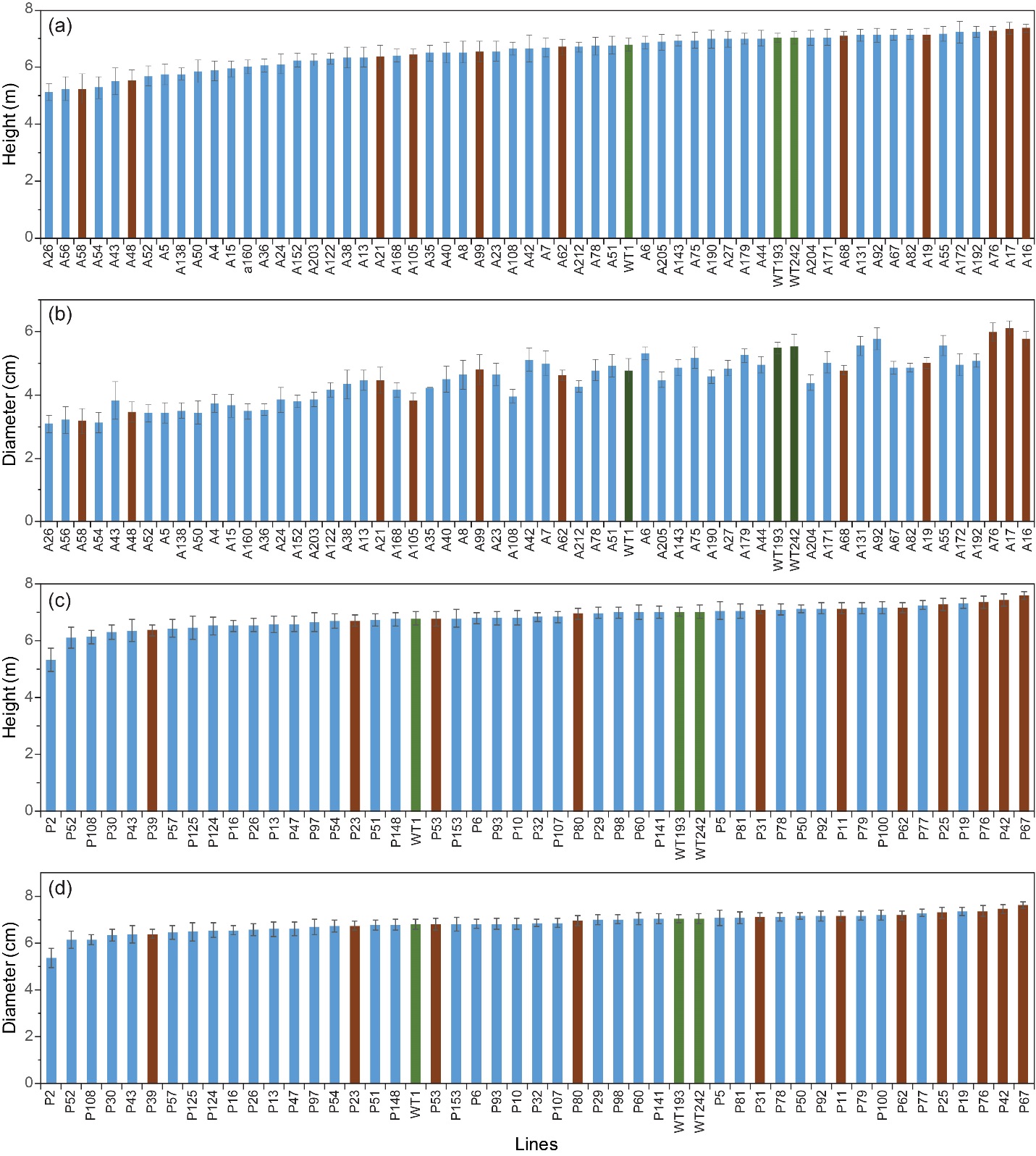
**

**Figure S5.** Stem heights and diameters of transgenic *LtCAAT1*/*LtAPS1* (a, b) and *LtCAAT1*/*LtPPS1* (c, d) hybrid poplar lines, as well as that of WT. Lines shown in red were sampled in August 2014, whereas WT trees sampled then are shown in green. For each line, 20 trees were measured for height and diameter.


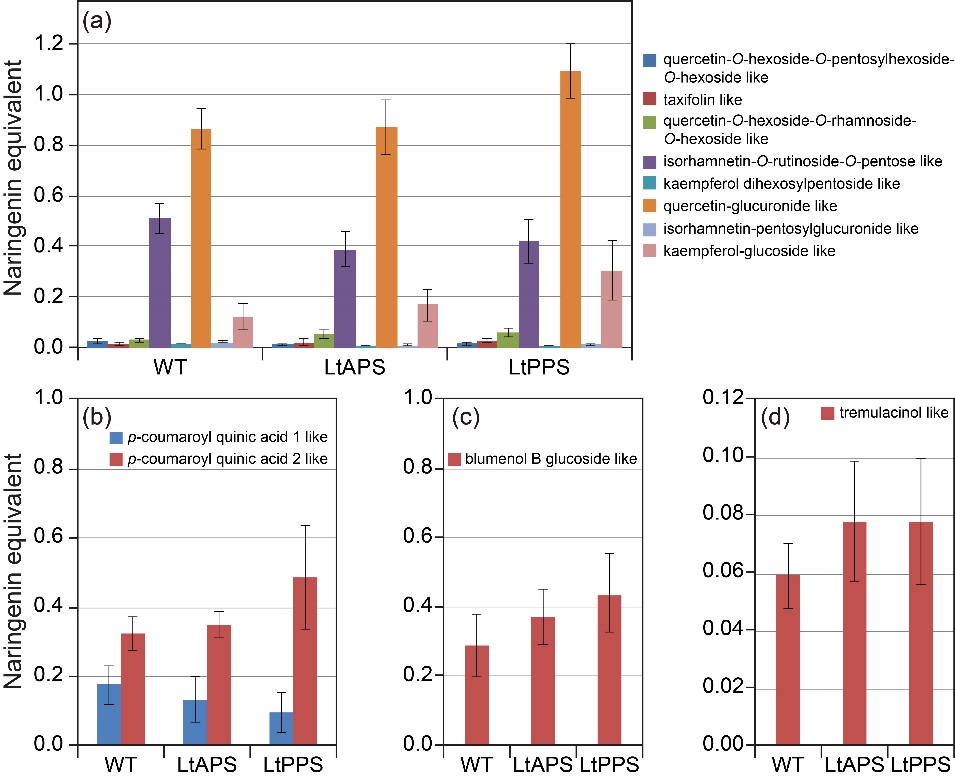


**Figure S6**. Putative metabolites detected by non-targeted UPLC metabolomic analyses of WT and transgenic LtAPS and LtPPS lines. Putative flavonoid like (a), hydroxycinnamyl quinate derivative like (b), blumenol like (c), and tremulacinol like (d) metabolite level changes between WT, LtAPS and LtPPS lines..


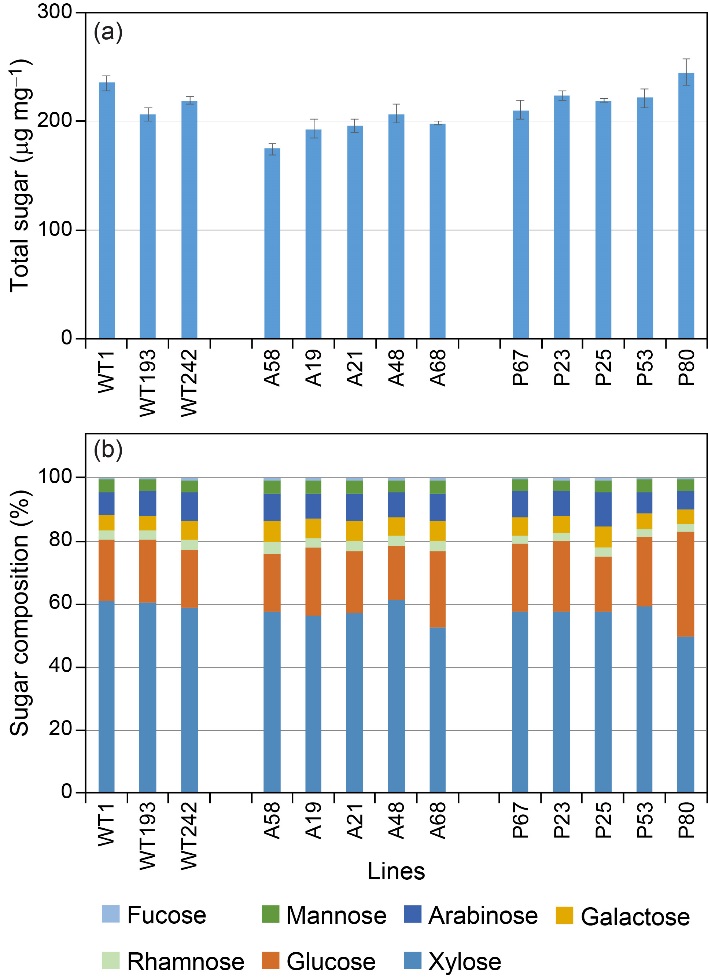


**Figure S7.** Matrix polysaccharide content (a) and composition (b) in branches of hybrid poplar WT and those expressing LtAPS and LtPPS.

**
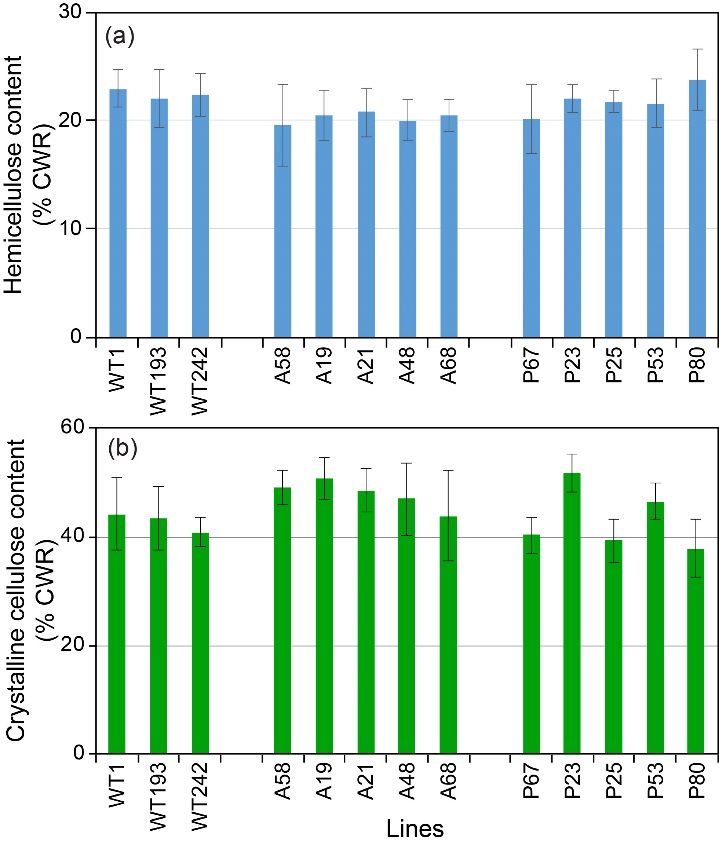
**

**Figure S8**. Hemicellulose (a) and crystalline cellulose (b) contents in branches of hybrid poplar WT and those expressing LtAPS and LtPPS.

**
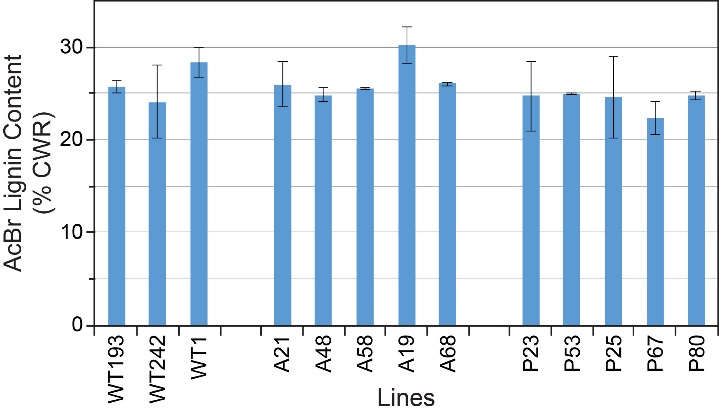
**

**Figure S9**. Acetyl bromide lignin estimations in branches of hybrid poplar WT and those expressing LtAPS and LtPPS.

**
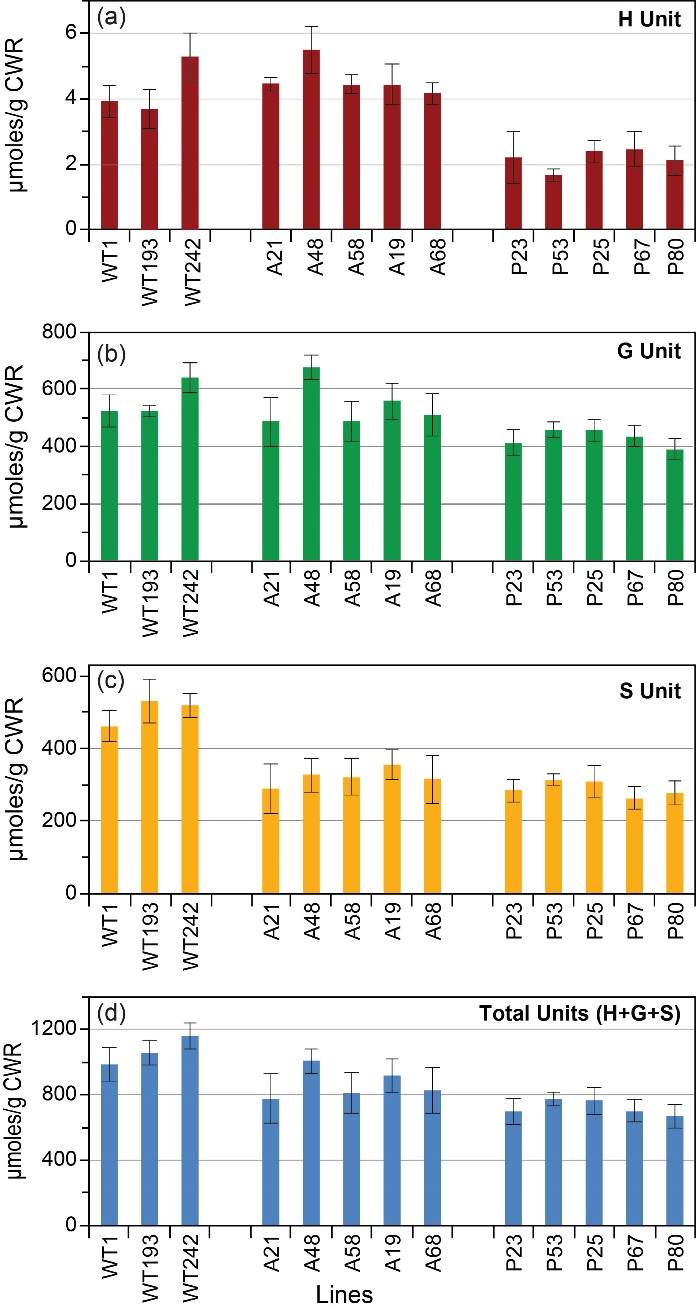
**

**Figure S10.** Estimated lignin compositions by thioacidolysis in branches of hybrid poplar (*P. tremula* × *P. alba*) WT and those expressing LtAPS and LtPPS. (a) *p*-hydroxyphenyl (H) unit. (b) Guaiacyl (G) unit. (c) Syringyl (S) unit. (d) Total units (H + G + S).
